# Comparison of variations detection between whole-genome amplification methods used in single-cell resequencing

**DOI:** 10.1186/s13742-015-0068-3

**Published:** 2015-08-06

**Authors:** Yong Hou, Kui Wu, Xulian Shi, Fuqiang Li, Luting Song, Hanjie Wu, Michael Dean, Guibo Li, Shirley Tsang, Runze Jiang, Xiaolong Zhang, Bo Li, Geng Liu, Niharika Bedekar, Na Lu, Guoyun Xie, Han Liang, Liao Chang, Ting Wang, Jianghao Chen, Yingrui Li, Xiuqing Zhang, Huanming Yang, Xun Xu, Ling Wang, Jun Wang

**Affiliations:** 1 BGI-Shenzhen, Shenzhen, 518083 China; 2 State Key Laboratory of Bioelectronics, School of Biological Science and Medical Engineering, Southeast University, Nanjing, 210096 China; 3 Cancer and Inflammation Program, National Cancer Institute at Frederick, Building 560, Frederick, MD 21702 USA; 4 BioMatrix, LLC, 3029 Windy Knoll Court, Rockville, MD 20850 USA; 5 Collage of Life Science, University of Chinese Academy of Sciences College, 19A Yuquan Road, Beijing, 100049 China; 6 Stanford University, 450 Serra Mall, Stanford, CA 94305 USA; 7 Department of Vascular and Endocrine Surgery, Xijing Hospital, Fourth Military Medical University, Xi’An, 710032 China; 8 The Guangdong Enterprise Key Laboratory of Human Disease Genomics, BGI-Shenzhen, Shenzhen, 518083 China; 9 Princess Al Jawhara Centre of Excellence in Research of Hereditary Disorders, King Abdulaziz University, Jeddah, 21589 Saudi Arabia; 10 James D Watson Institute of Genome Sciences, Zhejiang University, Hangzhou, 310058 China; 11 Department of Biology and the Novo Nordisk Foundation Center for Basic Metabolic Research, University of Copenhagen, Copenhagen, 1599 Denmark

**Keywords:** Whole genome amplification, Single-cell resequencing, Variations detection, DOP-PCR, MDA, MALBAC, Next-generation sequencing

## Abstract

**Background:**

Single-cell resequencing (SCRS) provides many biomedical advances in variations detection at the single-cell level, but it currently relies on whole genome amplification (WGA). Three methods are commonly used for WGA: multiple displacement amplification (MDA), degenerate-oligonucleotide-primed PCR (DOP-PCR) and multiple annealing and looping-based amplification cycles (MALBAC). However, a comprehensive comparison of variations detection performance between these WGA methods has not yet been performed.

**Results:**

We systematically compared the advantages and disadvantages of different WGA methods, focusing particularly on variations detection. Low-coverage whole-genome sequencing revealed that DOP-PCR had the highest duplication ratio, but an even read distribution and the best reproducibility and accuracy for detection of copy-number variations (CNVs). However, MDA had significantly higher genome recovery sensitivity (~84 %) than DOP-PCR (~6 %) and MALBAC (~52 %) at high sequencing depth. MALBAC and MDA had comparable single-nucleotide variations detection efficiency, false-positive ratio, and allele drop-out ratio. We further demonstrated that SCRS data amplified by either MDA or MALBAC from a gastric cancer cell line could accurately detect gastric cancer CNVs with comparable sensitivity and specificity, including amplifications of 12p11.22 (*KRAS*) and 9p24.1 (*JAK2*, *CD274*, and *PDCD1LG2*).

**Conclusions:**

Our findings provide a comprehensive comparison of variations detection performance using SCRS amplified by different WGA methods. It will guide researchers to determine which WGA method is best suited to individual experimental needs at single-cell level.

**Electronic supplementary material:**

The online version of this article (doi:10.1186/s13742-015-0068-3) contains supplementary material, which is available to authorized users.

## Background

Variations detection in single-cell resequencing (SCRS) research has enabled numerous advances in heterogeneity analysis [1], including cancer research [2–5], haplotype studies [6, 7], single-neuron sequencing [8], and detection of aneuploidy and unbalanced chromosomal rearrangement in pre-implantation screening/diagnosis [9, 10]. The direct sequencing of single cells has been limited by the picogram amount of DNA in individual cells; hence, whole genome amplification (WGA) is usually used to increase the amount of DNA before sequencing library preparation.

Currently, three WGA strategies are widely used for SCRS: degenerate-oligonucleotide-primed polymerase chain reaction (DOP-PCR) [11, 12], multiple displacement amplification (MDA) [13–15], and a combination of displacement pre-amplification and PCR amplification (marketed as PicoPlex kit by Rubicon Genomics [16, 17], MALBAC kit by Yikon Genomics [7, 18, 19]). These three WGA strategies differ in the enzymes used and in the experimental protocol design, which may yield different performances and biases, allowing for different specific applications. Quake et al. reported the comparison of CNVs detection, single-nucleotide variations (SNVs) detection and de-novo genome assembly using single-cell *Escherichia coli* DNA amplified by these three methods, with the corresponding bulk DNA as control [20]. He et al. compared the performance of genome coverage efficiency, reproducibility, GC bias, genome coverage uniformity and CNVs detection of 11 hippocampal neurons also amplified by these three methods at low-coverage sequencing depth [21]. Voet et al. reported the variations detection performance comparison using human cell line and blastomeres amplified by MDA and PicoPlex WGA [22].

However, although it is known that the WGA strategies may introduce artifacts and cause errors in variations detection [1], there is still no comprehensive comparison of the amplification bias and variations detection performance of the widely used commercialized kits completely based on these three strategies. To systematically evaluate the SCRS performance of commonly used WGA methods, we performed single-cell WGA using seven kits, with several experimental replicates for each kit, and then sequenced the whole genome of the successfully amplified DNA. We designed a narrowing-down strategy to investigate the amplification and variations detection performance cost-efficiently. First, we evaluated the mapping ratio, duplication ratio, and genome coverage uniformity using the single-cell low-coverage whole genome sequencing (LWGS) data or the extracted single-cell LWGS data. By evaluating the amplification quality during LWGS comparison, we selected the kits with best genome recovery sensitivity or uniformity. Using the further deep-sequenced whole genome sequencing (WGS) data amplified by the chosen kits, we further investigated the amplification bias and variations detection ability. In this way, we found that DOP-PCR methods had the highest duplication ratio and limited mapping efficiency and genome recovery - presumably as a result of the PCR process - but also that DOP-PCR methods had the best reproducibility and accuracy for detection of CNVs. In addition, we found that MDA and MALBAC had comparable genome recovery sensitivity, higher than that of DOP-PCR. Furthermore, we found that SCRS data from MDA also had comparable SNVs detection accuracy and CNVs detection accuracy to that of MALBAC. Our results provide a comprehensive comparison of variations detection performance at single-cell level between different WGA methods, and guidance for researchers to choose best suited WGA methods when performing variations detection at single-cell level.

## Data description

**Fig. 1:**
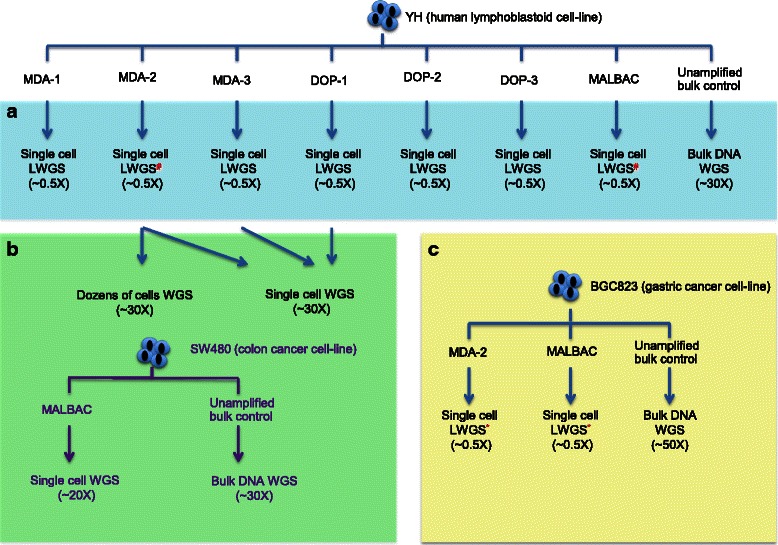
A narrowing-down strategy used to compare WGA methods cost-effectively. We describe the narrowing-down strategy using 3 panels (**a**, **b**, **c**). We perform LWGS comparison including genome coverage and uniform using YH single cells which are amplified by seven WGA kits based on DOP, MDA and MALBAC methods in panel A. We additionally compare the CNVs detection using simulated data of YH single cells in panel A. In panel B, we perform the deep WGS comparison of biases and SNVs detection using deep-sequenced YH or SW480 single cells amplified by DOP, MDA or MALBAC respectively. Corresponding bulk data is used as unamplified control. In panel C, we further compare the CNVs detection between MDA-2 and MALBAC amplified data using real data of BGC823 single cells. *Ion Proton sequencing data; #Illumina and Ion Proton sequencing data. LWGS, low-coverage whole-genome sequencing; WGS: whole genome sequencing. DOP-1,GenomePlex® Single Cell WGA Kit; DOP-2, Silicon Biosystem Ampli*™* WGA Kit; DOP-3, NEB Single Cell WGA Kit; MDA-1, Qiagen REPLI-g Mini Kit; MDA-2, Qiagen REPLI-g Single Cell Kit; MDA-3, GE Healthcare illustra GenomiPhi V2 DNA Amplification Kit; MALBAC,Yikon Genomics Single Cell Whole Genome Amplification Kit. Data marked in purple is downloaded

As shown in Fig. 1, we used a narrowing-down strategy to compare the WGA methods cost-effectively. We obtained 29 single cells from the YH cell line (a human lymphoblastoid cell line from first Asian genome donor [23]) and amplified them using seven commercialized kits. The kits tested were: GenomePlex® Single Cell WGA Kit (which we called DOP-1, Sigma-Aldrich, St. Louis, MO, USA); Silicon Biosystem Ampli*™* WGA Kit (DOP-2, Silicon Biosystems, Bologna, Italy); NEB Single Cell WGA Kit (DOP-3, New England Biolabs, Ipswich, MA, USA); Qiagen REPLI-g Mini Kit (MDA-1, Qiagen, Düsseldorf, Germany); Qiagen REPLI-g Single Cell Kit (MDA-2, Qiagen, Düsseldorf, Germany); GE Healthcare illustra GenomiPhi V2 DNA Amplification Kit (MDA-3, GE Healthcare, Little Chalfont, Buckinghamshire, England); and Yikon Genomics Single Cell Whole Genome Amplification Kit (MALBAC, Yikon Genomics, China). These kits were based on DOP-PCR, MDA, or MALBAC method respectively as indicated by their designations. We performed several experimental replicates for each kit, and sequenced the WGA product of each single cell a mean depth of ~0.5X (Additional file 1: Table S1 and Additional file 2: Table S2). We performed a low-coverage sequencing comparison using 20 YH single cells which were amplified by these seven WGA kits and sequenced them on Illumina Sequencer (Additional file 1: Table S1). Three out of the 20 YH single cells that showed outstanding uniformity during low-coverage sequencing comparison and two other YH single cells amplified by MDA-2 kit were also selected to further high-coverage sequence to around 30X on Illumina Sequencer (Additional file 3: Table S3). We also obtained deep WGS data from two sets of YH cells (each set was comprised of 10–20 single YH cells) whose DNA was amplified using the MDA-2 kit (called MDA-2_M6 and MDA-2_M16; Additional file 3: Table S3). We obtained the bulk WGS data from the YH cell line as control (called YH-mix; Additional file 3: Table S3). Seven other YH single cells were amplified by MDA-2 or MALBAC, then sequenced on Lifetech Ion Proton Sequencer to perform CNVs detection (Additional file 2: Table S2).

For the cancer cell line data, we downloaded the MALBAC-amplified WGS data of five single cells derived from the SW480 human colon cancer cell line and corresponding bulk SW480 sequencing data from the NCBI Short Read Archive (SRA060929).

Finally, we obtained 10 single cells from a human gastric cancer cell line (called BGC823), amplified five by MALBAC and five by MDA-2, and sequenced them to ~0.5X depth on Lifetech Ion Proton Sequencer. We also obtained the WGS data of the bulk DNA of BGC823 as a control (Additional file 2: Table S2).

## Analyses

### Comparison of low-coverage single-cell WGS performance

**Fig. 2:**
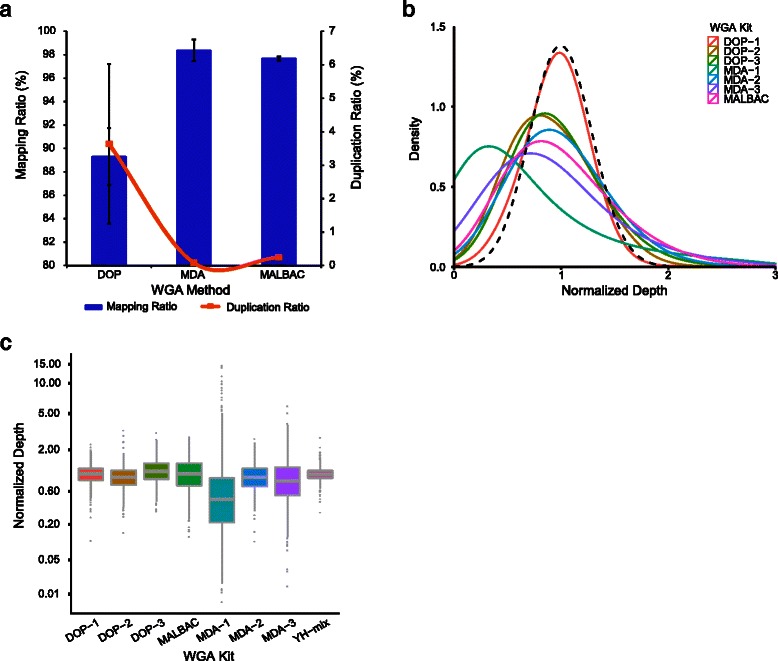
LWGS Comparison of recovery sensitivity and amplification uniformity between WGA methods. **a** The recovery sensitivity comparison of three different WGA methods using 0.1X randomly extracted LWGS data. The histogram and line graph show the mean mapping ratio and mean duplication ratio of different methods, respectively. **b** The mean normalized depth distribution of the seven WGA kits using the 0.1X sequencing data. The normalized read depth is defined as the ratio of the mean depth of all reads in each window to the mean depth of the whole genome. The binning window is 100 kb. The dashed curve is plotted using the simulated data (1000 dots) that followed the Poisson distribution (λ = 30) and normalized by dividing by 30. **c** A comparison of mean normalized depth distribution in chr15:q11.1-q26.3 between different WGA kits. The binning window is 100 kb. YH-mix is used as the unamplified control

We first aligned the raw short reads of 20 low-coverage sequenced YH single cells to the human reference genome (hg19) using BWA [24] (Methods). The resulting data, including the read mapping ratio, read duplication ratio, GC content, depth, and genome coverage, was summarized and evaluated in Additional file 1: Table S1. To eliminate the impact of sequencing depth and sequencer bias on the WGA comparison, we randomly extracted 0.1X data from the raw LWGS data (Additional file 4: Table S4). We found that MDA-2 amplified data had the highest mean genome coverage (8.84 %), even higher than that of MALBAC (8.06 %). MDA and MALBAC amplified data had lower duplication ratio than DOP-PCR amplified data (Bonferroni-corrected Mann–Whitney-Wilcoxon test, *p* < 0.05), but higher mean mapping ratio than DOP amplified data (Average 98.36 %, SD 0.92 % for MDA; average 97.68 %, SD 0.17 % for MALBAC; and average 89.31 %, SD 2.41 % for DOP, Bonferroni-corrected Mann–Whitney-Wilcoxon test, *p* < 0.05) (Fig. 2a).

To gain more insights into the distinction of the mapping ratio between different WGA methods, we then investigated unmapped reads for their GC content, sequencing quality, and mapping quality. We found no significant difference in the GC content of unmapped reads between the methods (Additional file 5: Figure S1). However, we found a significantly different N ratio for the unmapped reads among the three WGA methods, with that for MALBAC being the highest (Bonferroni-corrected Mann–Whitney-Wilcoxon test, *p* < 0.001) and that for MDA being the lowest (Bonferroni-corrected Mann–Whitney-Wilcoxon test, *p* < 0.001) (Additional file 6: Figure S2). The lowest N ratio seen in MDA-amplified data could be explained by the high fidelity of the Phi29 polymerase. Also, the different amplification primers and the different sequencing quality may cause the N ratio distinction, either.

We compared the read distribution uniformity using 0.1X extracted data from all the YH single cells mentioned above. We simulated the theoretic sequencing depth distribution which followed the Poisson distribution (1000 dots, λ = 30) and was normalized by dividing by 30. We then found that the mean normalized sequencing depth distribution of DOP-1 data was most similar with the theoretic one, whereas all other amplification kits had observed bias (Fig. 2b). Overall, the mean normalized depth distribution biases for DOP-PCR methods, MDA-2 and MALBAC were lower than those of MDA-1 and MDA-3. DOP-PCR and MALBAC showed higher reproducibility than MDA (Additional file 7: Figure S3, Additional file 8: Table S5, Bonferroni-corrected Mann–Whitney-Wilcoxon test, *p* < 0.05).

Using the 0.1X extracted data, we then assessed the regional reads distribution in one genomic region in which there were no copy-number alterations in the YH-mix data (chr15: q11.1-q26.3). The read distribution for DOP-PCR, MDA-2, and MALBAC had better evenness and reproducibility than other WGA kits, and the MDA-1 read distribution demonstrated the highest bias (Bonferroni-corrected Mann–Whitney-Wilcoxon test, *p* < 0.001), as also found in a previous report [18] (Fig. 2c, Additional file 9: Figure S4 and Additional file 10: Figure S5).

In summary, SCRS data amplified by MDA or MALBAC had a lower duplication ratio, a higher mapping ratio, and a higher genome recovery than that from DOP-PCR. DOP-PCR, MDA-2 and MALBAC amplified data showed high uniformity and reproducibility. All three amplification strategies could potentially provide a uniform distribution of sequencing reads, which is important for CNVs analysis at the single-cell level.

### Deep single-cell WGS and bias evaluation

To further explore the genome coverage bias introduced by WGA, we compared deep-sequenced data (~30×) amplified by DOP-1, MDA-2, MDA-3 or MALBAC respectively (Table 1), because LWGS data amplified by these four WGA kits had better genome recovery sensitivity or sequence evenness than other kits. Among 5 deep-sequenced YH single cells, MDA-3 amplified data covered more than 94.35 % of the reference genome, and mean genome coverage of 3 MDA-2 amplified single cells was 97.72 % (SD 2.97 %). We downloaded deep-sequenced data of five SW480 single cells (derived from a colon cell line) amplified by MALBAC cells from previous report [18], which covered a mean of 82 % (SD 9.42 %) of the whole genome. Of note, DOP-1 amplified YH WGS data covered only 23.23 % of the reference genome with sequencing depth ~30X (we received ~30X raw sequencing data, and after removing the primer sequences and duplications we obtained ~3X mapping reads with DOP-1). The low amplification efficiency of the DOP-PCR method, which resulted from the random primer PCR and the enzyme [12], may cause the high duplication ratio (39.24 %) at the whole genome level.

**Table 1:** Deep-sequencing statistics of single cells amplified by different kits

Sample index	Number of mapped bases (bp)	Read mapping ratio (%)	Read duplication ratio (%)	GC content (%)	Mean depth (X)	Genome coverage (%)
MDA-2_46	109,113,164,019	98.42	2.44	43.63	38.20	94.30
MDA-2_47	82,746,143,862	98.49	1.73	42.74	28.95	99.63
MDA-2_66	102,165,179,471	98.54	6.52	40.66	35.84	99.24
MDA-3_45	52,911,771,602	99.09	6.17	39.40	18.52	94.35
DOP-1_97	8,294,107,956	86.18	39.24	40.65	3.00	23.23
SW480-1	55,385,452,648	94.34	7.50	42.95	19.45	91.33
SW480-2	57,344,758,117	94.69	7.51	42.86	20.15	91.63
SW480-3	66,569,935,382	93.54	19.64	40.40	23.42	83.33
SW480-4	78,746,822,579	92.56	21.83	39.91	27.76	70.88
SW480-5	40,966,360,470	89.53	7.05	40.36	14.50	74.87
SW480-HEC	104,576,495,349	96.49	3.82	42.84	36.59	99.01
SW480-SCD	88,079,534,311	91.39	3.43	39.42	30.99	99.13
YH-mix	109,269,489,080	95.97	10.77	41.39	38.30	99.68

**Fig. 3:**
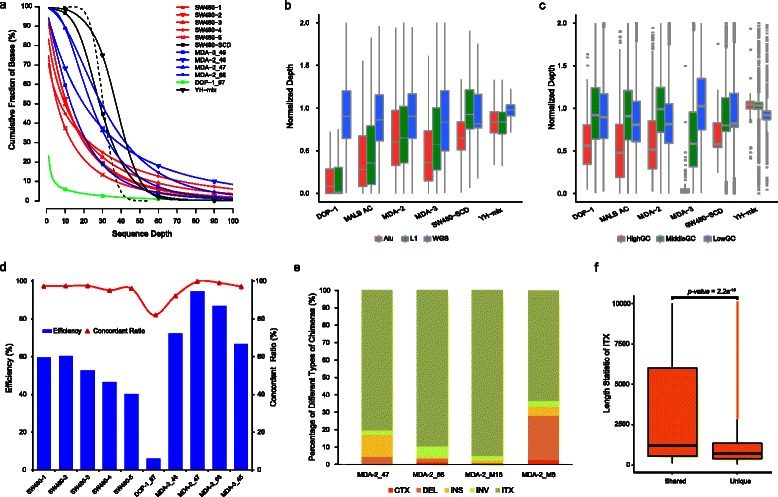
Bias and chimeras comparison using WGS data. **a** The cumulative distribution of sequencing fold depth of deep WGS data amplified by DOP-1, MDA-2, MDA-3, and MALBAC, respectively. The standard Poisson Cumulative Distribution (λ = 30) is plotted (dashed), and YH-mix and SW480 bulk data are presented as a control. It was related to Additional file 11: Table S6. **b** Normalized read depth distribution in repeat regions (Alu and L1 regions) and the entire genome of deep-sequenced data amplified by different WGA kits. The normalized read depth is calculated for each Alu/L1 region and for each window binning 100 kb of the entire genome. **c** Normalized read depth distribution in regions with different GC content of deep-sequenced data amplified by different WGA kits. The 100 kb windows with GC content >50 % are defined as ‘HighGC’ windows, <35 % as ‘LowGC’ windows, and others as ‘MiddleGC’ windows. **d** Histogram of effective consensus genotype efficiency, and line graph of the concordant ratio of all deep-sequenced cells amplified by different WGA kits compared to the golden control. **e** The percentage of different types of chimeras detected in MDA-2-amplified YH single cells. CTX, inter-chromosomal translocation; ITX, intra-chromosomal translocation; DEL, deletion: INS, insertion: INV, inversion. **f** Boxplot of the length distribution of ITXs shared between MDA-2-amplified cells and YH-mix versus the chimera ITXs that are unique in single cells. *p* < 0.01, Mann–Whitney-Wilcoxon test

We next determined the cumulative sequencing depth distribution across the entire genome to evaluate amplification bias. Cumulative depth distribution curves for DOP-1, MDA-2, MDA-3, and MALBAC fitted a standard Poisson distribution (Fig. 3a). Although the YH-mix and bulk SW480 control datasets covered almost whole reference genome with a sequencing depth of 10X or more (98.62 % and 96.65 %, respectively), the coverage of WGA data was much lower – average 82.21 % (SD 11.98 %) in MDA-2, 59.49 % in MDA-3, 5.93 % in DOP-1, and average 47.33 % (SD 6.32 %) in MALBAC, respectively. All three WGA strategies therefore introduced amplification bias, but MDA-2 showed the highest effective covered sequencing depth that may best suited for variations calling (Bonferroni-corrected Mann–Whitney-Wilcoxon test, *p* < 0.001, Additional file 11: Table S6).

To further determine the specific regional bias and GC bias introduced by WGA, we next used the deep-sequenced data to evaluate the normalized depth distribution in Alu and L1 repeat regions and regions with different GC content. We plotted the distributions of normalized depth in each Alu and L1 region, compared with the distribution in entire genome split into 100 kb windows. We observed that the normalized depth distribution of DOP-1 amplified data in Alu and L1 regions was significantly lower than that at whole-genome level, and DOP-1 amplified data had the greatest difference of normalized depth distribution between the repeat regions and whole genome among different WGA methods (Fig. 3b, Bonferroni-corrected Mann–Whitney-Wilcoxon test, *p* < 0.001). In addition, the read distribution of SCRS data from DOP-1 was influenced slightly by GC content, as the result of the unamplified control of YH-mix (Fig. 3c), whereas high GC content influenced the read distribution of the MDA-3 data (Bonferroni-corrected Mann–Whitney-Wilcoxon test, *p* < 0.001).

Using the deep-sequenced data, we performed extra comparison of assembly performance between MDA and MALBAC amplified data, and found that MALBAC may have comparable assembly quality as MDA but lower stability of the assembly than MDA by mitochondrial assembly (See details in Additional file 12: Supplementary Note).

### Assessment of artifacts introduced by different WGA methods

To gain more comprehensive insights into the single-nucleotide artifacts introduced by the three amplification methods, we first defined a ‘golden control’ genotype set for MDA and DOP-PCR amplified data: a set of genotype consensus sites from the YH-mix that were also found on the 2.5 M Illumina Omni SNP Chip (Methods). We also defined a ‘golden control’ genotype set for MALBAC: shared genotype consensus sites between bulk sequencing data of SW480-SCD and SW480-HET that were also found on the 2.5 M Illumina Omni SNP Chip. We compared the consensus genotypes from the DOP-1, MDA-2, MDA-3, and MALBAC deep-sequenced data with the corresponding golden controls (Table 2 and Additional file 13: Table S7), and evaluated the consensus genotypes detection efficiency (CGDE) and concordant ratio (Methods). The mean CGDE of MDA-2 data was 84.57 % (up to 94.62 %) , and the mean concordant ratio was 97.10 % (up to 99.88 %). By contrast, data from DOP-1, MDA-3, and MALBAC sequencing had a substantially lower CGDE (6.00 %, 66.63 %, and a mean of 51.87 %, respectively), with concordant ratio of 82.05 %, 97.12 % and a mean of 96.74 %, respectively (Fig. 3d). The limitations of CGDE indicated a common WGA bias in these different methods; however, data from MDA-2 had less bias.

**Table 2:** Comparison of consensus genotypes and SNVs detection accuracy of deep-sequenced data amplified by MDA and MALBAC

	Allele type		Golden control for SW480 cells				
			HOM ref.	HOM mut.	HET ref.	Total	Consistency (%)
			1,762,437.00	403,431.00	173,098.00	2,338,966.00	
MALBAC mean	HOM ref.	2	849,057.40	-	-		
		1	-	-	10,352.40	859,455.20	98.79
		0	-	45.40	-		
	HOM mut.	2	-	266,889.00	-		
		1	-	-	18,507.40	285,625.60	93.44
		0	213.40	11.20	4.60		
	HET ref.	2	-	-	58,948.80		
		1	2,287.20	6,860.40	8.80	68,105.20	86.56
		0	-	0.00	-		
	Total		851,558.00	273,806.00	87,822.00	1,213,186.00	96.84
	Coverage (%)		48.32	67.87	50.74	51.87	-
	Allele type		Golden control for YH cells				
			HOM ref.	HOM mut.	HET ref.	Total	Consistency (%)
			1,584,649.00	270,225.00	351,490.00	2,206,364.00	
MDA mean	HOM ref.	2	1,373,228.00	-	-		
		1	-	-	21,871.00	1,395,113.33	98.43
		0	-	14.33	-		
	HOM mut.	2	-	256,682.67	-		
		1	-	-	27,674.00	284,365.67	90.26
		0	7.33	1.67	0.00		
	HET ref.	2	-	-	256,185.67		
		1	212.67	326.33	2.33	256,727.00	99.79
		0	-	0.00	-		
	Total		1,373,448.00	257,025.00	305,733.00	1936,206.00	97.41
	Coverage (%)		86.67	95.12	86.98	87.76	-

Mean coverage and consistency are calculated using the data amplified by the same WGA method according to Additional file 13: Table S7. HOMref, homozygotes where both alleles are identical to the reference; HOMmut, homozygotes where both alleles are different from the reference; HETref, heterozygotes where only one allele is identical to the reference. We formulate the mean counts of genotyped alleles of single cell sequencing sites that are consistent with ‘golden control’ at both alleles, at one allele, or that are inconsistent at both alleles as 2, 1, and 0, respectively

To further investigate the potential biological impact of these discordant genotypes sites (present in single cells but not in the golden control) in SCRS data introduced by the WGA, we sorted out the discordant SNVs among these discordant genotype sites in three deep-sequenced single cells amplified by MDA-2, and then annotated these discordant SNVs using ANNOVAR [25] (Additional file 14: Table S8). We found that most of the altered genes that contained discordant SNVs occurred only in one of the three cells, and only ~ 4 % of the altered genes were shared among all of the three cells (Additional file 15: Figure S6), indicating that the artifacts introduced by the MDA-1 were unlikely to influence the gene category analysis.

Because MDA frequently introduced chimeras [26], we used deep WGS data of two single YH cells (MDA-2_47 and MDA-2_66) and another two sets of 10–20 single YH cells (MDA-2_M6 and MDA-2_M16, Additional file 3: Table S3) to evaluate the amplification chimeras. We performed breakpoints identification using CREST [27] in these samples as well as YH-mix as a control (Methods). We defined the chimeras as the breakpoints appeared only in the single-cell data rather than in YH-mix. Of the different types of breakpoints such as the insertion (INS), deletion (DEL), inversion (INV), intra-chromosomal translocation (ITX) and inter-chromosomal translocation (CTX), we found that chimeric ITX (Additional file 16: Figure S7) was the dominant chimera type (82.08 %, Fig. 3e). In addition, we found a significant difference of length distribution between true ITXs (shared by the YH-mix) and chimeric ITXs in single cells (Fig. 3f), suggesting that the chimeras tended to be produced by neighboring amplicons randomly connecting on the same chromosome, as previously reported [26]. The percentages of other chimera types, such as chimeric CTX (Additional file 17: Figure S8), deletion, insertion and inversion, were 1.13 %, 8.09 %, 5.07 %, and 3.68 %, respectively.

### Single-cell SNVs and CNVs detection accuracy of the WGA methods

Owing to the amplification bias discussed above, SCRS may lose one or both alleles at specific genome loci during amplification (we termed sites with the loss of one allele ‘allele drop-out’ sites, ADO) (Methods). In addition, WGA may introduce additional alleles that might lead to false-positive mutations at the single-cell level (we termed sites with WGA-introduced alleles as false-positive sites, FP). The high duplication ratio and low genome coverage of DOP-PCR methods limited their application in SNVs detection; so we just compared the SNVs detection accuracy of MDA with MALBAC (downloaded data from the previous reports [18]). Taking the YH-mix as the golden control for MDA-2 amplified data, we calculated the SNVs detection accuracy of deep-sequenced single cells amplified by MDA-2, and showed the results in Table 3. We detected a mean of 3,044,473 SNVs in MDA-2 amplified single cells compared with 3,649,573 SNVs in the YH-mix, thus the overall detection efficiency for the MDA-2 data was 83.42 %. We then calculated the ADO ratio and FP ratio of SCRS data amplified by MDA-2, founding that the mean ADO ratio was 12.47 % (0.78 %, 3.23 % and 33.40 %, respectively), and the mean FP ratio was 5.31 × 10^−5^ (SD 0.007 %) which was comparable with ~4 × 10^−5^ of the FP ratio of MALBAC in previous report [18]. Although the 5.31 × 10^−5^ false-positive ratio for SNVs detection (due to the amplification enzyme) appeared to be a problem for accurately genotyping single-cell whole human genomes (3 × 10^9^ sites), by integrating the consensus sequence of two or three independent cells, the false-positive ratio could be decreased to ~10^−8^ with two replicate cells and to ~10^−12^ with three replicate cells, as described in previous report [18]. In conclusion, we inferred that the SCRS data generated using MALBAC and MDA-2 had a comparable performance for SNVs detection, with up to ~10^−12^ false positives across the entire genome of a single cell.

**Table 3:** Comparison of consensus genotypes and SNVs detection accuracy of deep-sequenced data amplified by MDA and MALBAC

Control/sample	Heterozygous (FP/ADO/Efficiency)	Homozygous (FP/Efficiency)	Total (FP/Efficiency)	FP ratio	ADO Ratio
YH-mix (Unamplified control)	2051,282	1,598,291	3,649,573	-	-
MDA-2_46	777,908 (5563/390,038/37 %)	1,747,004 (390,107/84 %)	2,524,912 (395,670/58 %)	1.32E-04	0.3340
MDA-2_47	1,807,282 (6517/14,124/87 %)	1,562,036 (14,177/96 %)	3,369,318 (20,694/91 %)	6.90E-06	0.0078
MDA-2_66	1,651,733 (6347/55,158/80 %)	1,587,456 (55,195/95 %)	3,239,189 (61,542/87 %)	2.05E-05	0.0323

**Fig. 4:**
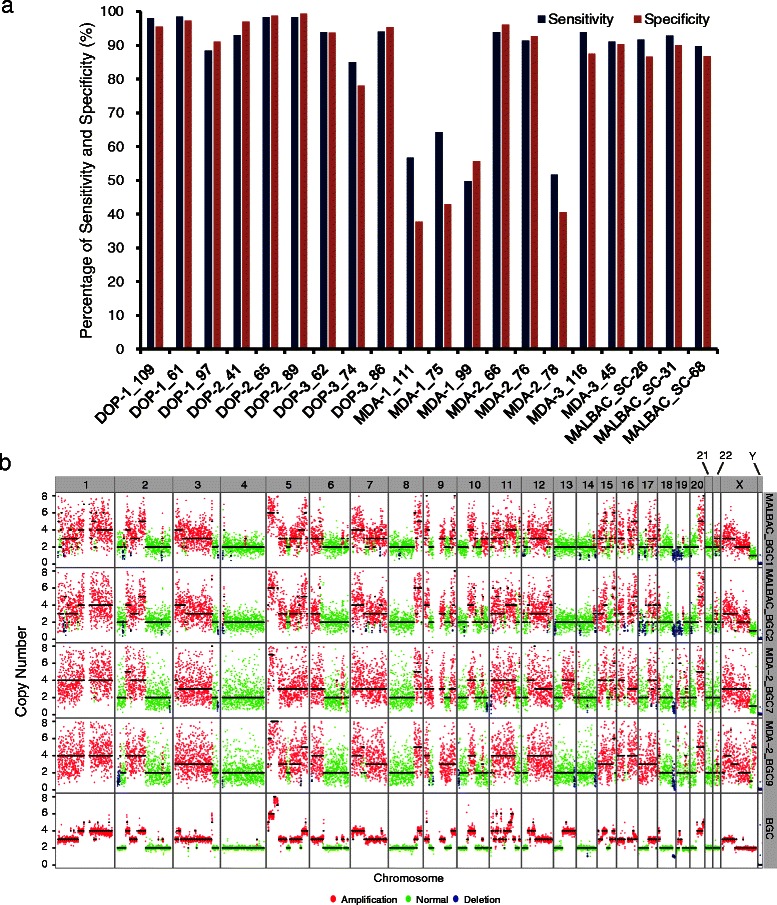
Read-data CNVs detection comparison between MALBAC and MDA-2 amplified data. **a** Taking the simulated YH-mix data as control, sensitivity and specificity of CNVs (≥1 Mb) in simulated single YH cells amplified by different WGA methods are bar-plotted. **b** CNVs of BGC823 single cells amplified by MALBAC or MDA-2. BGC823 single cells are sequenced on the Ion Proton sequencer (~0.5X) as control. Bulk BGC823 sequencing data (bottom row) are sequenced by PE-100 on an Illumina Hiseq 2000 (~50X), and ~1X data was extracted randomly to detect CNVs. Green, red, and blue represent normal, amplification, and deletion, respectively

To systematically compare the performance of the SCRS data from MALBAC, MDA, and DOP-PCR for CNVs detection, we first used the 0.1X LWGS data described in Additional file 4: Table S4 to compare the CNVs detection accuracy (Methods). Because the YH cell line was derived from normal lymphocytes with few CNVs (≥1 Mb), we only observed few large CNVs at the single-cell level (Additional file 18: Figure S9) in most of the SCRS data amplified with DOP-PCR, MALBAC or MDA. To further compare the CNVs detection of different WGA method using these data, we simulated some CNVs candidates into each single YH cell data and YH-mix data (Methods). These CNVs candidates were determined as the concordant CNVs of the SW480-SCD bulk data and the SW480-HET bulk data. We then called CNVs using a pipeline modified from Baslan’s method [28] (Methods). Comparing each single cell data with the YH-mix data as control, we found that DOP-PCR had the best accuracy for CNVs detection (≥1 Mb, Bonferroni-corrected Mann–Whitney-Wilcoxon test, *p* < 0.05), with a mean sensitivity of 94.15 % (SD 4.84 %) and a mean specificity of 94.00 % (SD 6.51 %). Simulated data from MALBAC could detect CNVs (≥1 Mb) with a mean sensitivity of 91.40 % (SD 1.61 %) and a mean specificity of 87.80 % (SD 1.98 %), whereas simulated SCRS data from MDA could detect CNVs (≥1 Mb) with only a mean sensitivity of 74.04 % (SD 20.21 %) and a mean specificity of 67.93 % (SD 25.97 %) (Fig. 4a; see also Additional file 19: Table S9 and Additional file 20: Figure S10). We found that although the mean CNVs detection sensitivity and specificity for the SCRS data from MDA was lower than those of DOP-PCR and MALBAC, the mean CNVs detection mean sensitivity and mean specificity for the SCRS data from MDA-2 were 78.91 % and 76.47 %, respectively, and one specific MDA-2 cell (cell MDA-2_66) even reached 93.84 % and 96.13 %, respectively. We also calculated the pair-wise Pearson correlation of copy-number ratio of all single cell data, and found that the SCRS data from MALBAC and DOP-PCR had significantly higher consistency than MDA, indicating that MALBAC and DOP-PCR have better reproducibility in CNVs detection (Additional file 21: Table S10, Bonferroni-corrected Mann–Whitney-Wilcoxon test, *p* < 0.05).

To further investigate the power of CNVs detection using real SCRS data amplified by MALBAC and MDA-2, we amplified 10 additional cells from a human gastric adenocarcinoma cell line (BGC823) using MALBAC (5 cells) and MDA-2 (5 cells) respectively, and sequenced them on Lifetech Ion Proton sequencer. BGC823 bulk sequencing data was used as the unamplified data control. We also introduced 7 YH single cells data which were amplified by MALBAC (3 cells) or MDA-2 (4 cells) and then sequenced on Lifetech Ion Proton sequencer. We found no recurrent CNVs (≥1 Mb) in the single YH cells and YH-mix, and did not identify any obvious different CNVs compared with YH cells sequenced on Illumina platform (Additional file 2: Table S2), indicating that different sequencing platforms (Illumina and Lifetech Ion Proton Sequencers) made few impacts on the CNVs detection comparison. We observed 213 major CNVs larger than 1 Mb in the bulk sequencing data of BGC823 (Additional file 22: Table S11), and most of the major CNVs in bulk sequencing data of BGC823 overlapped with CNVs in single BGC823 cells**,** including amplification regions that include the oncogene *KRAS* (12p11.22-p11.21) and the recently reported recurrent amplification at 9p24.1 at the locus containing *JAK2*, *CD274*, and *PDCD1LG2* (which augments the anti-tumor immune response) [29] (Fig. 4b, Additional file 23: Figure S11a, Additional file 24: Figure S11b and Additional file 22: Table S11). Treating the bulk sequencing data of BGC823 as control, we estimated that the MALBAC-amplified BGC823 SCRS data achieved a mean sensitivity of 84.72 % (SD 0.82 %) and a mean specificity of 85.18 % (SD 1.61 %), while MDA-2 amplified BGC823 SCRS data achieved a mean sensitivity of 85.86 % (SD 10.27 %) and a mean specificity of 81.18 % (SD 8.90 %), indicating that MALBAC provided a higher specificity and slightly lower sensitivity than MDA-2 (Additional file 25: Table S12). This result is different with our simulated data, which may be caused by difference in CNVs complexity between different cancer cell lines. In addition, MALBAC showed a higher reproducibility among replicates than MDA-2 in CNVs detection (Additional file 26: Table S13, Mann–Whitney-Wilcoxon test, *p* < 0.01), which is consistent with simulation data result. However, taking our findings on CNVs together, we concluded that the SCRS data from both MALBAC and MDA-2 could robustly identify CNVs larger than 1 Mb.

## Discussion

Here, we provided a comprehensive comparison of single-cell variations detection performance basing on different WGA methods. We first performed LWGS analysis of single cells using three major WGA methods: MDA, DOP-PCR, and MALBAC. The results indicated that SCRS data generated by MDA-2 (MDA using the Qiagen REPLI-g Single Cell Kit) presented higher genome recovery sensitivity than those generated by MALBAC and DOP-PCR with the same sequencing depth. SCRS data from DOP-PCR had the lowest amplification bias along the entire genome, as well as high reproducibility and the highest single-cell CNVs detection accuracy (>90 %). In contrast to previous reports [18], our analysis showed that MDA-2 and MALBAC had similarly favorable detection accuracy and efficiency for single-cell SNVs and CNVs detection, although MDA and MALBAC introduced FP sites, ADO sites, and amplification bias.

DOP-PCR based sequencing data showed high duplication ratio and limited genome recovery sensitivity in our study, indicating that this method may not be suitable for detecting additional SNVs and structural variations at deep sequencing depth. However, DOP-PCR has also been reported to accurately detect aneuploidy and unbalanced chromosomal rearrangements, achieving 99.63 % sensitivity and 97.71 % specificity for detecting CNVs larger than 1 Mb [30]. Considering our result together, we suggest that DOP-PCR methods are suitable for studies focusing on the analysis involving number of sequencing reads, such as CNVs or aneuploidy detection in pre-implantation screening/diagnosis, cancer research or other disease research.

We found that MALBAC sequencing data had intermediate genome recovery sensitivity, and uniformity for CNVs detection. A previous study showed that MALBAC was advantageous for SNVs and CNVs detection in SCRS data compared with MDA (based on the kit called MDA-1 here) [18]. However, when we compared the SNVs and CNVs detection performance of the MDA-2 kit (an optimized version of the MDA-1 kit), we found that the MDA-2 data had higher genome recovery than the MALBAC data with the same sequencing depth (Additional file 4: Table S4, Additional file 27: Table S14). More importantly, we found that the MDA-2 data had a comparable SNVs detection accuracy and CNVs detection accuracy with those of the MALBAC data; and this accuracy was greater than that indicated by a previous report for MDA-1 [18]. Taken together, these data suggest that optimization of MDA experimental protocols may significantly improve SNVs and CNVs detection in SCRS data. Thus, we conclude that both MDA and MALBAC can be used for research that require low duplication ratio and high genome coverage, for example, the detection of SNVs in disease research. In addition, if researchers need to perform SNVs and CNVs detection at the same time in some fields like tumor heterogeneity and evolution research, we recommend using MDA-2 and/or MALBAC because of their higher efficiency and accuracy in variations detection. However, MALBAC may have higher reproducibility of uniformity and of CNVs detection performance than MDA-2, making MALBAC more conducive to the heterogeneity research related to variations detection.

Although MDA method would introduce chimeras during WGS, our analysis indicated that chimeras of MDA-2 had potential to detect the breakpoints of structural variations for specific types of structural variations at the single-cell level, such as inter-chromosomal structural variations, with the possibility of increasing the specificity by reducing the number of random chimeras in an increasing number of replicate cells.

A remaining challenge for variations detection at the single-cell level is the cost. Unlike bulk sequencing, single-cell analysis needs to amplify the whole genome of the single cell first. The cost, especially for a large number of cells to be amplified before sequencing, will be considerable when taking the failure ratio into consideration. The MDA and DOP-PCR are the most widely used WGA methods even before the single-cell sequencing occurs, and their costs are relatively low, especially if using homemade reagents following the freely available protocol. However, MALBAC is a new method with more complex experimental procedure that was developed especially for single-cell sequencing, and thus the cost will be higher than that of MDA and DOP. We believe that more detailed published protocols and more users will help further reduce the cost of MALBAC for single-cell amplification. Another approach that may reduce the cost significantly for all three amplification methods could be microfluidics, which would limit the reaction into a very small volume (several nanoliters) for a large number of amplified single cells [31].

Our results provide a comprehensive comparison of variations detection performance in SCRS with different WGA methods. It will guide researchers to choose the most optimal WGA method to perform specific single cell sequencing project in research areas such as analysis of circulating tumor cells and tumor evolution, and pre-implantation screening and diagnosis.

## Methods

### Sample preparation before WGA

A total of 39 single cells were collected in our study, 29 from a lymphoblastoid cell line (YH cell line) established from the first Asian genome donor [23], the rest from a widely known gastric cancer cell line, BGC823. Corresponding bulk DNA was extracted as an unamplified control. The BGC823 cell line was provided by Youyong Lv at Beijing Cancer Hospital. All samples and experimental protocols were approved by the Institutional Review Board of BGI-Shenzhen.

Single cells were isolated as described previously [3]. Briefly, following sufficient dissociation and dilution of cells, single cells were randomly picked up using a mouth pipette under a microscope and washed three times in phosphate-buffered saline to avoid exogenous DNA contamination, then transferred into a PCR tube. Single-cell isolation was confirmed by microscopy to ensure that only one cell was inside each tube.

### WGA of single-cell genomic DNA with different WGA methods

WGA was performed using seven different commercial kits based on MDA, DOP-PCR or MALBAC strategies. The kits used were Qiagen REPLI-g Mini Kit (MDA-1), Qiagen REPLI-g Single Cell Kit (MDA-2), GE Healthcare illustra GenomiPhi V2 DNA Amplification Kit (MDA-3), GenomePlex® Single Cell WGA Kit (DOP-1), Silicon Biosystem Ampli1™ WGA Kit (DOP-2), NEB Single Cell WGA Kit (DOP-3), and Yikon Genomics Single Cell WGA Kit (MALBAC). All experimental operations followed the manufacturers’ protocols strictly and without any modification.

### Library construction and whole-genome DNA sequencing

The Illumina sequencer and LifeTech Ion Proton sequencer were used as the sequencing platforms in this study. To construct the library for each cell on the Illumina platform, 1–2 μg amplified genomic DNA was used. After fragmentation, the ‘A’ adaptor was ligated to each fragment. Next, 10 cycles of PCR using 8-base barcode primers was performed. After the DNA concentration and insert size measurement, the libraries were processed for paired-end high-throughput sequencing on Illumina HiSeq2000/HiSeq2500/MiSeq sequencer with a mean depth of ~ 0.5X. Libraries with outstanding performance in either recovery sensitivity or evenness of low-coverage sequencing were further deeply sequenced to around 30X. For LifeTech Ion Proton sequencing, a Bioruptor instrument was used to fragment DNA. The desired size of DNA fragments were obtained and ligated with Ion Proton A and P1 adaptors at each end, and then selected using E-Gel EX 2 % Gel (Invitrogen, Carlsbad, CA) for 150- to 200-bp fragments. The fragments were amplified, and the DNA was purified with Agencourt AMPure XP beads (Beckman Coulter Genomics, High Wycombe, UK). As assessed by the BioAnalyzer High Sensitivity LabChip Agilent, the resulting library had a median fragment size of 180 bp. After dilution, emulsion PCR reactions were set up for each nanoball in the library. Before the nanoballs were placed onto the ION PI chip, a sequencing primer and polymerase were added to the final enriched spheres.

### Read processing and mapping

#### Paired-ended reads generated by Illumina sequencer

Published data [18] of SW480 SCRS data and bulk SW480 sequencing data were downloaded from the NCBI Short Read Archive with accession no. SRA060929. The WGA primer was trimmed by Trimmomatic [32] from the 5′ ends of each read: 30 bases for YH cells amplified by DOP-PCR [11, 12] and 35 bases for SW480 and YH cells amplified by MALBAC [7, 18, 19]. Reads of YH cells amplified by MDA [13–15] did not need to be trimmed. Reads were then mapped to the human genome reference (Hg19, Build37) by BWA [24] (version 62) and SAMtools [33] (version 0.1.18), and sorted and marked as duplicates by Picard [34] (version 1.72). 0.1X data was then randomly down-sampled from the alignment results by Picard for each sample.

#### Single-ended reads generated by LifeTech Ion Proton sequencer

Thirty five bases of WGA primer were trimmed by Trimmomatic from the 5′ ends of each read of BGC and YH single cells amplified by MALBAC [18]. Reads were then mapped to the human genome reference (Hg19, Build37) by TMAP [35] (version 3.6.40) and SAMtools (version 0.1.18), and sorted and marked as duplicates by Picard (version 1.72).

The alignment result was checked for quality by Qualimap [36] (version 0.6).

### SNVs calling

For each deeply sequenced sample, low-quality alignments (mapping quality less than 1, unmapped, duplicates, and non-unique) were filtered using BamTools [37]. Filtered alignments were then processed by GATK [38] (version 2.3–9) with the options ‘Local Realignment around Indels’ and ‘Base Quality Score Recalibration’. SNVs were called at any callable sites by UnifiedGenotyper (a variation caller of GATK), and trained by a Gaussian mixture model using GATK. All the low-quality SNVs and false-positive SNVs were identified and then filtered based on the log odds ratio under the Gaussian mixture model.

### Excluding sequencing errors

In the LWGS study, we only used the Illumina sequencing data to perform the comparison. The sequencing error rates from the Illumina Miseq and Hiseq sequencer have been reported previously [39]. In the comparison, we directly mapped the sequencing reads to the hg19 human reference genome using BWA [24] with mismatches allowed. As with most variations calling that used resequencing data, we did not correct the sequencing errors of the raw sequencing reads; instead, we excluded the low-quality reads, sorted the mapping data and directly calculated the mapping ratio. To evaluate the bias in the comparison caused by the correctable sequencing errors from Hiseq and Miseq, we extracted the same amount of the sequencing reads amplified by the same kit but sequenced on Hiseq 2000 or Miseq, respectively. We found that there was no significant difference in the mapping ratio or duplication ratio between the cells sequenced by the Hiseq 2000 or Miseq (Additional file 28: Table S15). Thus, we inferred that the conclusions we generated from the LWGS data were not significantly biased by the correctable sequencing errors.

In the deep WGS study, the correctable sequencing errors may greatly influence the SNVs calling in the comparison. To exclude this influence introduced by the sequencing errors, we performed the following steps in the SNVs calling performance comparison between MDA and MALBAC:

1. For each deeply sequenced sample, low-quality alignments (mapping quality less than 1, unmapped, duplicates, and non-unique) were filtered using BamTools [37];
2. Alignments were processed by GATK [38] (version 2.3–9) with the options ‘Local Realignment around Indels’ and ‘Base Quality Score Recalibration’;
3. SNVs were called at any callable sites by UnifiedGenotyper (a Bayesian model based variation caller of GATK), and trained by a Gaussian mixture model using GATK (this step filtered out the influence of the sequencing errors and the mapping errors);
4. All the low-quality SNVs and false-positive SNVs were identified and then filtered based on the log odds ratio under the Gaussian mixture model;
5. The ADO ratio and false-positive ratio were calculated, by comparing the genotypes of single cells with those of the corresponding bulk sequencing data sequenced on the same sequencer. In this way, after the SNVs calling and filtering by the Bayesian model and Gaussian mixture model, we ensured that the sequencing errors did not bias the comparison results.

### Single-nucleotide artifacts analysis

We defined different ‘golden controls’ for different cell type data. For the YH single cells, the ‘golden control’ was defined as the concordant genotypes set overlapped between YH-mix data and a commercial 2.5 M Illumina Omni SNP Chip. And for the SW480 single cells, we first obtained an overlap set of concordant genotypes between the two SW480 bulk (SW480-SCD and SW480-HEC) sequencing data to reduce sequencing errors, and defined the ‘golden control’ as the intersection set of genotypes between the ‘overlap set’ and the commercial 2.5 M Illumina Omni SNP Chip. We clustered the genotyped alleles of both the ‘golden control’ and corresponding single cells into three categories: HOMref (homozygotes where both alleles were identical to the hg19 reference genome), HOMmut (homozygotes where both alleles were different with the hg19 reference genome), and HETref (heterozygotes where only one allele was identical to the hg19 reference genome). We formulated the counts of genotyped alleles of single cell sequencing sites that were consistent with ‘golden control’ at both alleles, at one allele, or that were inconsistent at both alleles as 2, 1, and 0, respectively.

For each category (HOMref, HOMmut and HETref), we calculated the consensus genotypes detection efficiency (CGDE) as the ratio of counts of consensus genotypes detected in single cell to those detected in corresponding control. Concordant ratio was defined as the ratio of counts of genotypes which both alleles were identical to the golden control to the genotypes detected in single cell for each category. We then calculated the mean CGDE and concordant ratio of all categories for each single cell.

### SNVs detection efficiency, ADO, and false-positive ratio calculation

SNVs detection efficiency was calculated as the ratio of the count of detected SNVs in a given single cell (minus the number of false-positive SNVs) to those in the bulk DNA. The ADO was defined as the non-amplification occurred in alternative alleles present in heterozygous sites. The false positive was defined as the SNVs in single cell sequencing data but not present in the bulk sequencing data. Both the ADO and false positive ratio were calculated by comparing the single cell sequencing data with bulk control sequencing data.

### Analysis of the chimera effect

To identify the chimeras at single-cell level, we identified breakpoints using CREST [27] both in the MDA-2 amplified samples and YH-mix. Taking the YH-mix as the control, the true breakpoints in MDA-2 amplified samples were defined as those overlapped with YH-mix if they were of the same type and were not further apart than a threshold of 100 bp: the rest were considered as chimeras.

#### CNVs simulation on the YH samples in silicon

Shared regions (≥1 Mb) between SW480-SCD and SW480-HEC with concordant CNVs (the copy number was assumed to be N) were selected as candidate regions for further CNVs simulation. The copy number ratio (assumed to be R) of the candidate region was formulated as the copy number of the region divided by 2 (*R* = N/2). For each YH sample (YH single cells and YH-mix control data), the simulated reads count (Ks) was defined as the product of the reads count of a bin (Kr) and the copy number ratio (R) of the corresponding candidate region. (Ks = Kr × R). The modified pipeline was then used to call CNVs in the simulated data for each sample.

### Data simulation and CNVs calling

Copy numbers were computed for each sample separately using a modified method based on that developed by the Cold Spring Harbor Laboratory [28]. Briefly, we performed following steps to detect CNVs:

1. Simulated single-ended reads (50 bp) from hg19 were mapped to hg19 by bowtie [40] (version 1.0.0). 10,000 genomics bins were used in the analysis.
2. Reads from the 0.1X LWGS alignments (BAM format) were converted to FASTQ format through the single-ended mode by BEDTools [41], and then re-mapped to hg19 reference genome by bowtie. Bases were trimmed from the 5′ end of each read to ensure that each read was 50 bp long. Raw reads generated by Lifetech Ion Proton sequencer (BAM format) were converted to FASTQ format by BEDTools, and then were trimmed by Trimmomatic to an effective length (50 bp plus length of WGA primer) from the 3′ end of the reads. The resulting alignments were re-mapped to hg19 reference genome by bowtie, and then bases were again trimmed from the 5′ end of each read to ensure each read was 50 bp long.
3. For each sample, segments were detected by DNAcopy [42], a circular binary segmentation (CBS) algorithm based CNVs detection tool. The density of the segment ratio of all bins within autosomes was plotted, and the mode of the segment ratio was set corresponding to a copy number of two.
4. Sensitivity and specificity were calculated (following [30]) as following:

$$ \mathrm{Sensitivity} = {\mathrm{L}}_{\mathrm{T}}/{\mathrm{L}}_{\mathrm{C}} $$

$$ \mathrm{Specificity} = {\mathrm{L}}_{\mathrm{T}}/\mathrm{L}, $$

where L represents the total length of CNVs (≥1 Mb) of a single cell detected by this pipeline, L_C_ represents the length of CNVs (≥1 Mb) of the corresponding control data (simulated YH-mix data) detected by this pipeline, and L_T_ represents the length of the region that the CNVs (≥1 Mb) of the single-cell overlap with the CNVs (≥1 Mb) of the corresponding control data.

#### Simulation on the YH genome in silicon

Shared regions (≥1 Mb) between SW480-SCD and SW480-HEC with concordant CNV (the copy number was assumed to be N) were selected as candidate regions for further CNV simulation. The copy number ratio (assumed to be R) of the candidate region is the copy number of the region divided by 2 (*R* = N/2). For each of the YH samples, to get the simulated reads count (Ks), the reads count of a bin (Kr) was multiplied by the copy number ratio (R) of the corresponding candidate region. (Ks = Kr × R). The modified pipeline was then used to call CNVs in the simulated data for each sample.

### Statistical analyses

We performed the Mann–Whitney-Wilcoxon test to assess the variations in cases of comparisons between two groups. Pearson correlations were calculated to investigate the similarity between metrics. To control the family-wise error rate, we performed the Bonferroni correction when multiple comparisons were conducted simultaneously.

## Availability of supporting data

The raw sequence data in the FASTQ format from previous reports [18] is available in the NCBI Short Read Archive repository [SRA060929]. The raw data in the fastq format, and the alignments and genotyping data from this study are hosted in the *GigaScience* Repository, GigaDB [43].

## Additional files

Additional file 1: Table S1. Summary of LWGS of YH single cells amplified by different WGA methods on Illumina sequencer. Additional file 2: Table S2. Summary of LWGS of BGC823 and YH single cells on Lifetech Ion Proton sequencer. # represented ~1× extracted data downsampled from the ~50× bulk BGC823 sequencing data (PE-100, Illumina Hiseq 2000) as unamplified control. Additional file 3: Table S3. Summary of deep WGS of YH single cells. YH-mix is used as the unamplified control. Additional file 4: Table S4. A comparison of recovery sensitivity between WGA methods using randomly extracted 0.1X data. Additional file 5: Figure S1. A comparison of GC content distributions of unmapped reads between different WGA methods. We calculate the GC content of each unmapped read and box-plotted the distributions for each WGA method. YH-mix data is plotted as the un-amplified control. Additional file 6: Figure S2. A comparison of the N ratio of unmapped reads between different WGA methods. We calculate the N ratio of each unmapped read and box-plot the distributions for each WGA method. YH-mix is used as the unamplified control. Additional file 7: Figure S3. The normalized depth distributions of all replicates. We plot the normalized read depth density distribution using the 0.1X extracted data. The normalized read depth is defined as the ratio of the mean depth of all reads in each window to the mean depth of the whole genome. The binning window is 100 kb. The dashed curve is plotted using simulated data (1000 dots) that followed the Poisson distribution (λ = 30) and normalized by dividing by 30. Additional file 8: Table S5. Pearson correlation of mean normalized depth between two replicates amplified by the same WGA kit. The binning window to assess the mean normalized depth is 100 kb. Additional file 9: Figure S4. Histograms of the mean depth distributions over a region of chr15 (20,000,001-102,521,388) for each kit. We calculate the mean depth of all replicates amplified with the same WGA kit at each site in the targeted region. YH-mix is used as the unamplified control. Additional file 10: Figure S5. Histograms of the depth distributions of all replicates over the same region of chr15 as Figure S4. YH-mix is used as the unamplified control. Additional file 11: Table S6. A comparison of genome coverage at different read depths between different WGA kits. YH-mix and SW480-SCD are used as unamplified controls. Additional file 12: **Supplementary Note.** The performance comparison of mitochondrial genome assembly between MDA and MALBAC. Additional file 13: Table S7. A comparison of consensus genotypes calling between different WGA kits. Additional file 14: Table S8. Genes annotation of all the discordant SNVs in the deep-sequenced single cells amplified by MDA-2. Additional file 15: Figure S6. Venn diagram of altered genes harboring discordant SNVs in 3 deep-sequenced cells amplified by MDA-2. Additional file 16: Figure S7. A schematic of one chimeric ITX calling within *WDHD1* gene in MDA-2_M6. The breakpoints and supporting reads of the chimeric ITX are shown. Additional file 17: Figure S8. A schematic of one chimeric CTX calling between chromosome 1 and 14 in MDA-2_47. The breakpoints and supporting reads of the chimeric CTX are shown. Additional file 18: Figure S9. The landscape of CNVs of YH single cells amplified by DOP, MALBAC, and MDA. The copy numbers across the genome are calculated for all YH single cells as well as for YH-mix using extracted ~0.1X data, and are then plotted. Green, red, and blue represent normal, amplification, and deletion, respectively. Additional file 19: Table S9. A sensitivity and specificity comparison of simulated CNVs detection between different WGA methods. Additional file 20: Figure S10. The landscape of simulated CNVs in YH single cells amplified by different WGA kits. YH-mix is used as the unamplified control. Additional file 21: Table S10. Pearson correlation of copy number ratios between simulated YH single-cell sequencing data. Additional file 22: Table S11. The list of CNVs (≥1 Mb) detected in the bulk BGC823 sequencing data. Additional file 23: Figure S11a. The landscape of CNVs of YH single cells amplified by MALBAC or MDA-2. These YH single cells were sequenced on the LifeTech Ion Proton sequencer, and YH-mix is used as the unamplified control. We extracted ~0.1X data from both the YH single cells and the mix to detect CNVs. Additional file 24: Figure S11b. The landscape of CNVs of BGC823 single cells amplified by MALBAC or MDA-2. These BGC823 single cells were sequenced on the LifeTech Ion Proton sequencer, and BGC823 bulk sequencing data is used as the unamplified control. We extracted ~0.1X data from both the BGC823 single cells and the bulk to detect CNVs. Additional file 25: Table S12. A sensitivity and specificity comparison of CNVs (≥1 Mb) between MALBAC and MDA-2. (XLSX 9 kb) Additional file 26: Table S13. Pearson correlation of copy number ratios between single cells amplified by MDA-2 or MALBAC kit. Additional file 27: Table S14. A comparison of the genome coverage between MDA-2 and MALBAC at 0.1X depth. The sequencing was performed on Lifetech Ion Proton sequencer. Additional file 28: Table S15. A comparison of the basic data sequenced on an Illumina Hiseq 2000 and a Miseq Sequencer. We extracted the same amount of the sequencing reads amplified by the same kit except the sequencing platform type to control the variables.

## Abbreviations

DOP-1: GenomePlex® Single Cell WGA Kit (St. Louis, MO, USA)
DOP-2: Silicon Biosystem Ampli*™* WGA Kit (Silicon Biosystems, Bologna, Italy)
DOP-3: NEB Single Cell WGA Kit (New England Biolabs, Ipswich, MA, USA)
MDA-1: Qiagen REPLI-g Mini Kit (Qiagen, Düsseldorf, Germany)
MDA-2: Qiagen REPLI-g Single Cell Kit (Qiagen, Düsseldorf, Germany)
MDA-3: GE Healthcare illustra GenomiPhi V2 DNA Amplification Kit (GE Healthcare, Little Chalfont, Buckinghamshire, England)
MALBAC: Yikon Genomics Single Cell Whole Genome Amplification Kit
ADO: Allele drop-out
CNVs: Copy number variations
DOP-PCR: Degenerate oligonucleotide-primed PCR
HETref: Heterozygotes where only one allele is identical to the reference
HOMmut: Homozygotes where both alleles differ from the reference
HOMref: Homozygotes where both alleles are identical to the reference
ITX: Intra-chromosomal translocations
LWGS: Low-coverage whole-genome sequencing
MALBAC: Multiple annealing and looping-based amplification cycles
MDA: Multiple displacement amplification
SCRS: Single-cell resequencing
SD: Standard deviation
SNVs: Single-nucleotide variations
WGA: Whole-genome amplification
WGS: Whole genome sequencing
