# Supplementary figures and images for: Comparison of variations detection between whole-genome amplification methods used in single-cell resequencing
Source: Gigascience. 2015 Aug 6;4:37. doi: 10.1186/s13742-015-0068-3 (PMC4527218; doi:10.1186/s13742-015-0068-3)

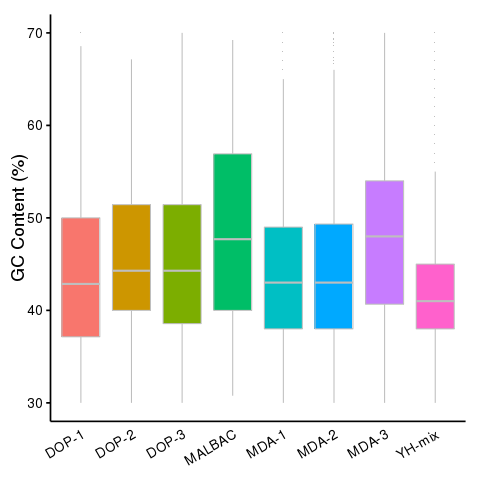

Supplement: Additional file 5: Figure S1. — A comparison of GC content distributions of unmapped reads between different WGA methods. We calculate the GC content of each unmapped read and box-plotted the distributions for each WGA method. YH-mix data is plotted as the un-amplified control. [file 13742_2015_68_MOESM5_ESM.png]

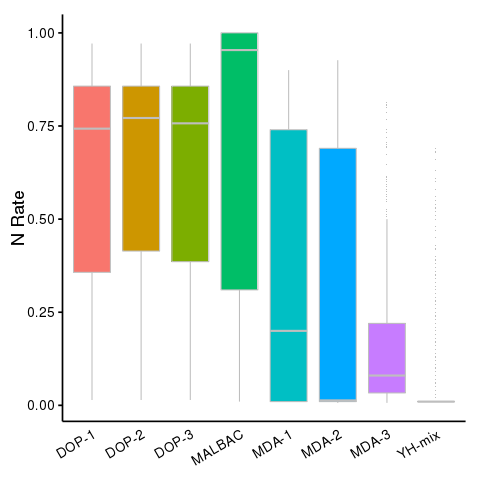

Supplement: Additional file 6: Figure S2. — A comparison of the N ratio of unmapped reads between different WGA methods. We calculate the N ratio of each unmapped read and box-plot the distributions for each WGA method. YH-mix is used as the unamplified control. [file 13742_2015_68_MOESM6_ESM.png]

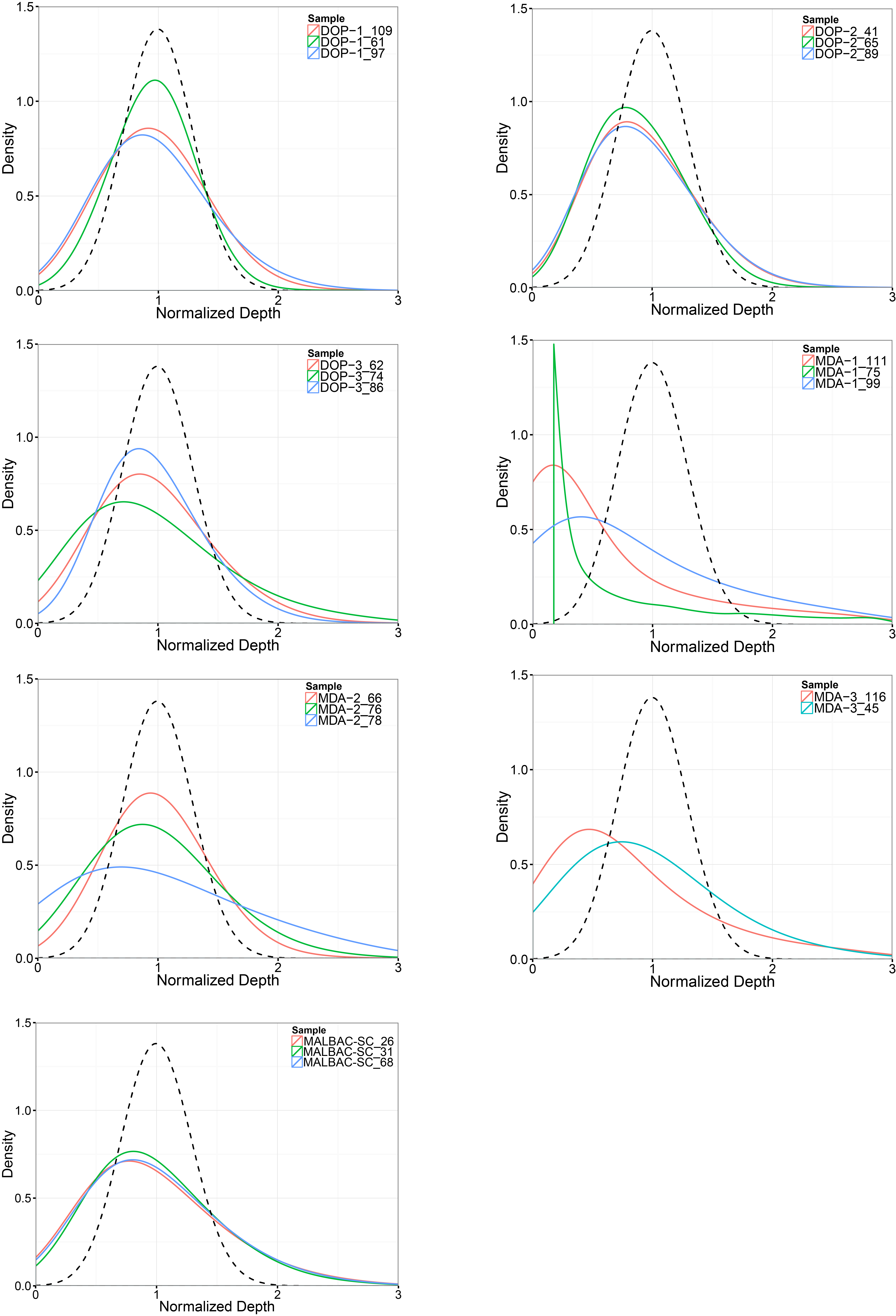

Supplement: Additional file 7: Figure S3. — The normalized depth distributions of all replicates. We plot the normalized read depth density distribution using the 0.1X extracted data. The normalized read depth is defined as the ratio of the mean depth of all reads in each window to the mean depth of the whole genome. The binning window is 100 kb. The dashed curve is plotted using simulated data (1000 dots) that followed the Poisson distribution (λ = 30) and normalized by dividing by 30. [file 13742_2015_68_MOESM7_ESM.png]

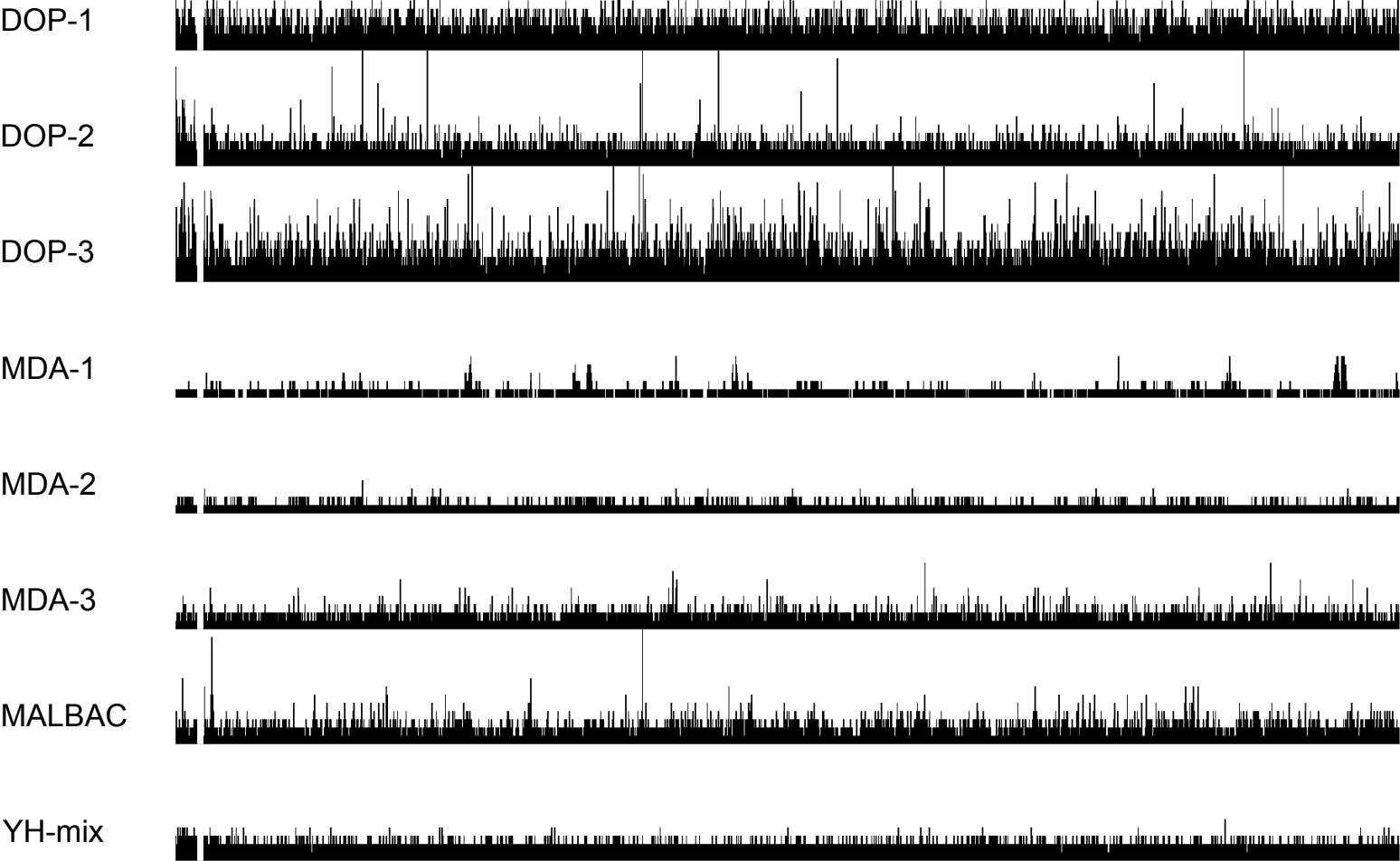

chr15 (q11.1-q26.3)

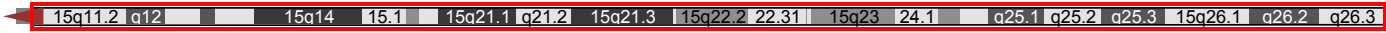

Supplement: Additional file 9: Figure S4. — Histograms of the mean depth distributions over a region of chr15 (20,000,001-102,521,388) for each kit. We calculate the mean depth of all replicates amplified with the same WGA kit at each site in the targeted region. YH-mix is used as the unamplified control. [file 13742_2015_68_MOESM9_ESM.pdf]

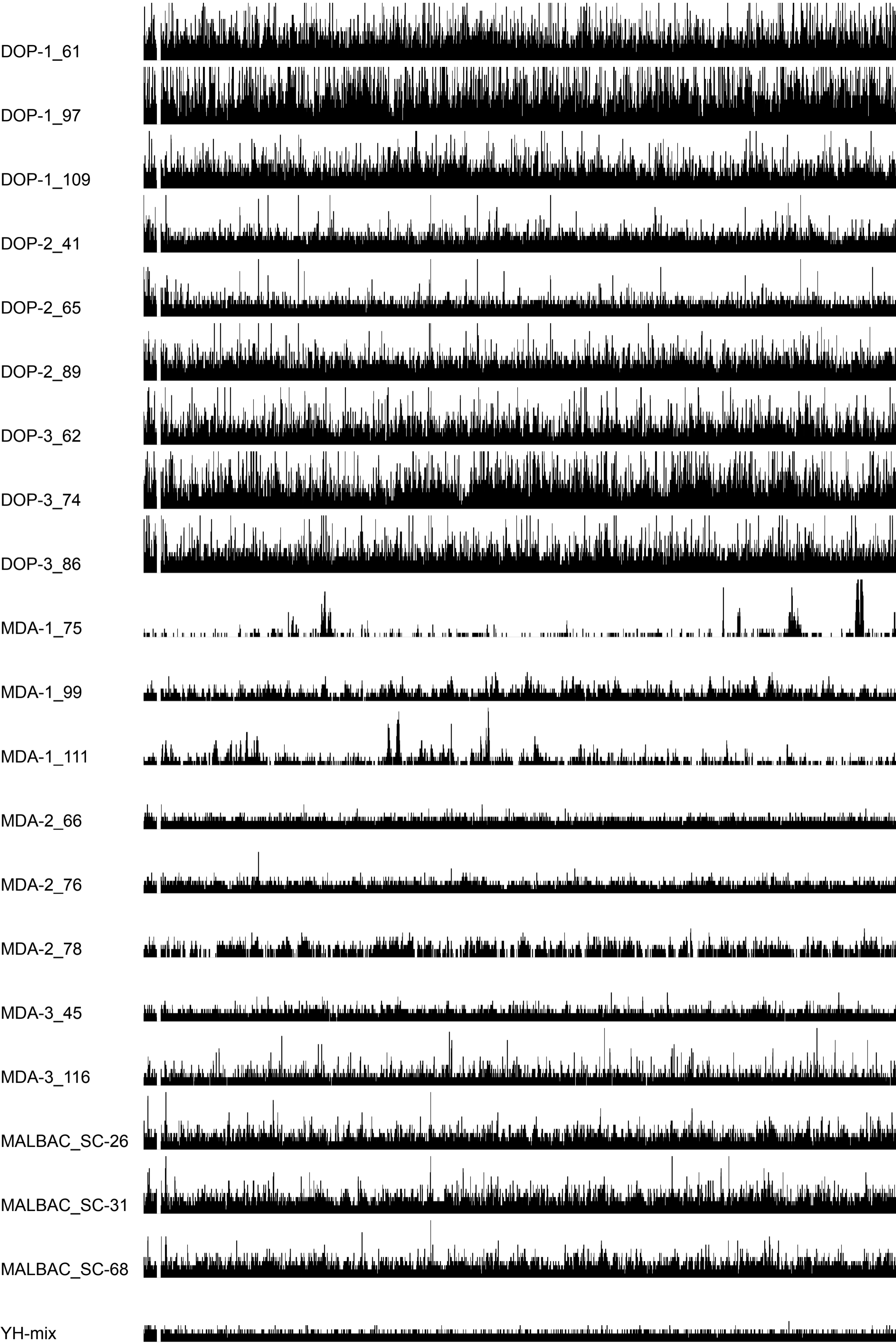

Supplement: Additional file 10: Figure S5. — Histograms of the depth distributions of all replicates over the same region of chr15 as Figure S4. YH-mix is used as the unamplified control. [file 13742_2015_68_MOESM10_ESM.pdf]

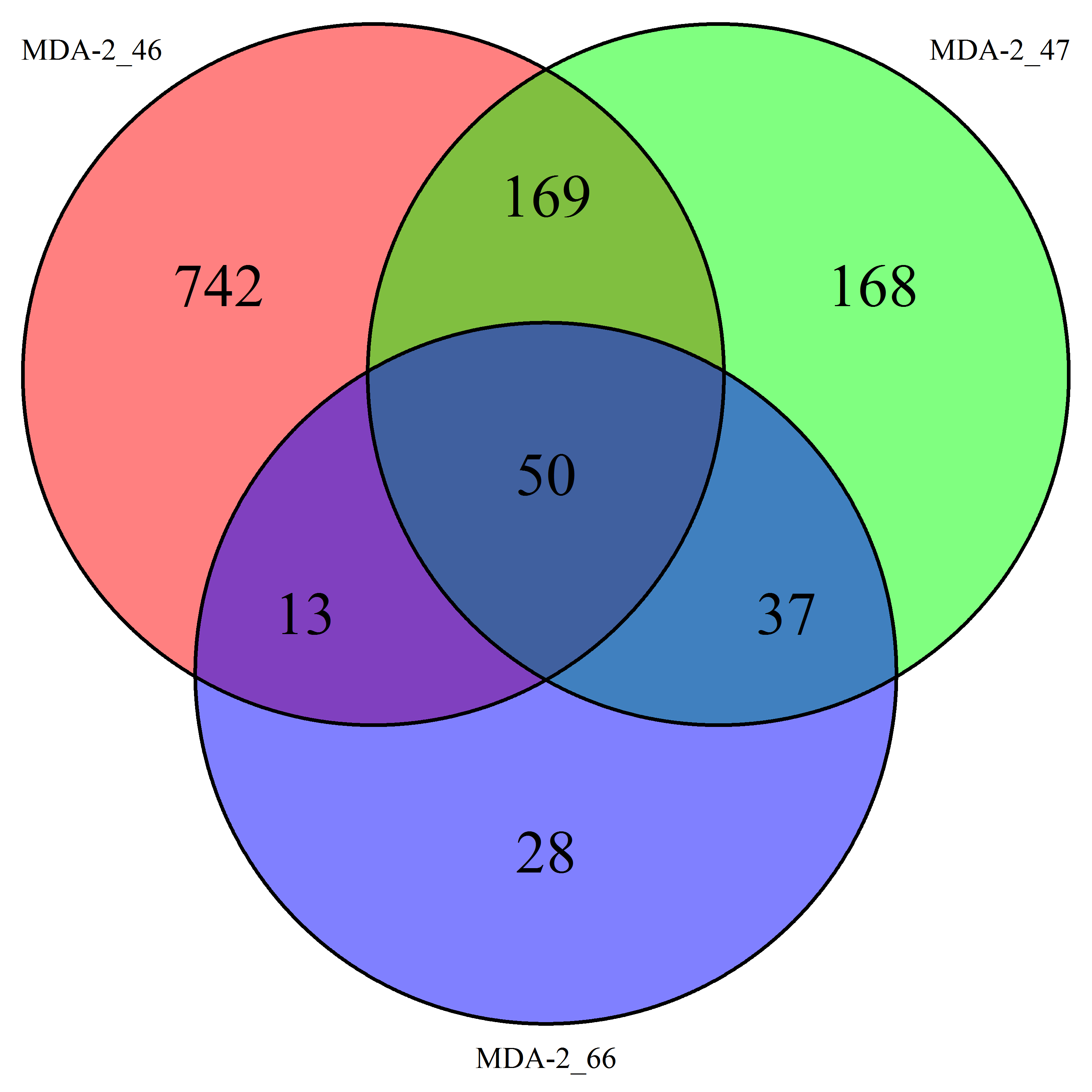

Supplement: Additional file 15: Figure S6. — Venn diagram of altered genes harboring discordant SNVs in 3 deep-sequenced cells amplified by MDA-2. [file 13742_2015_68_MOESM15_ESM.tiff]

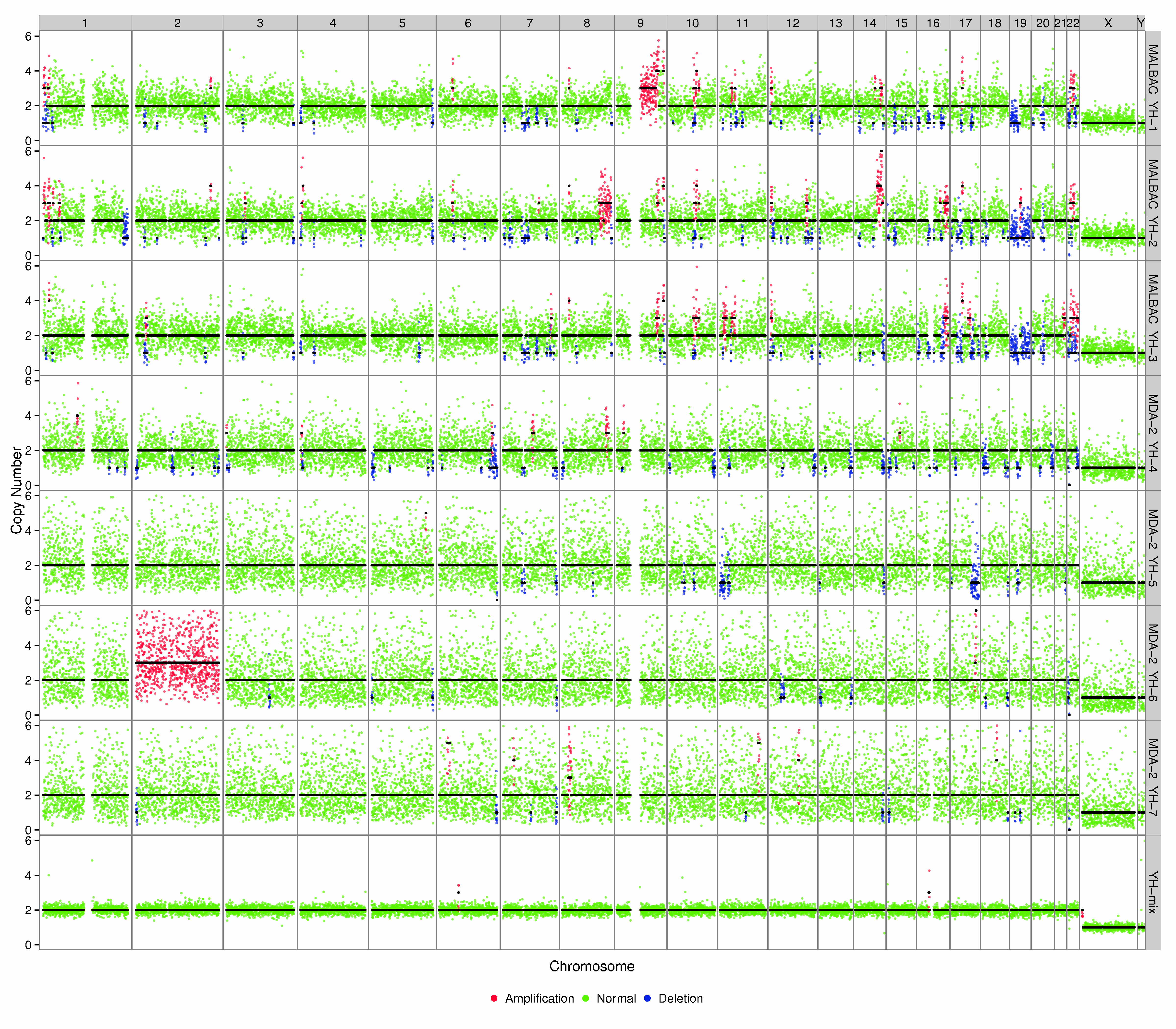

Supplement: Additional file 23: Figure S11a. — The landscape of CNVs of YH single cells amplified by MALBAC or MDA-2. These YH single cells were sequenced on the LifeTech Ion Proton sequencer, and YH-mix is used as the unamplified control. We extracted ~0.1X data from both the YH single cells and the mix to detect CNVs. [file 13742_2015_68_MOESM23_ESM.png]

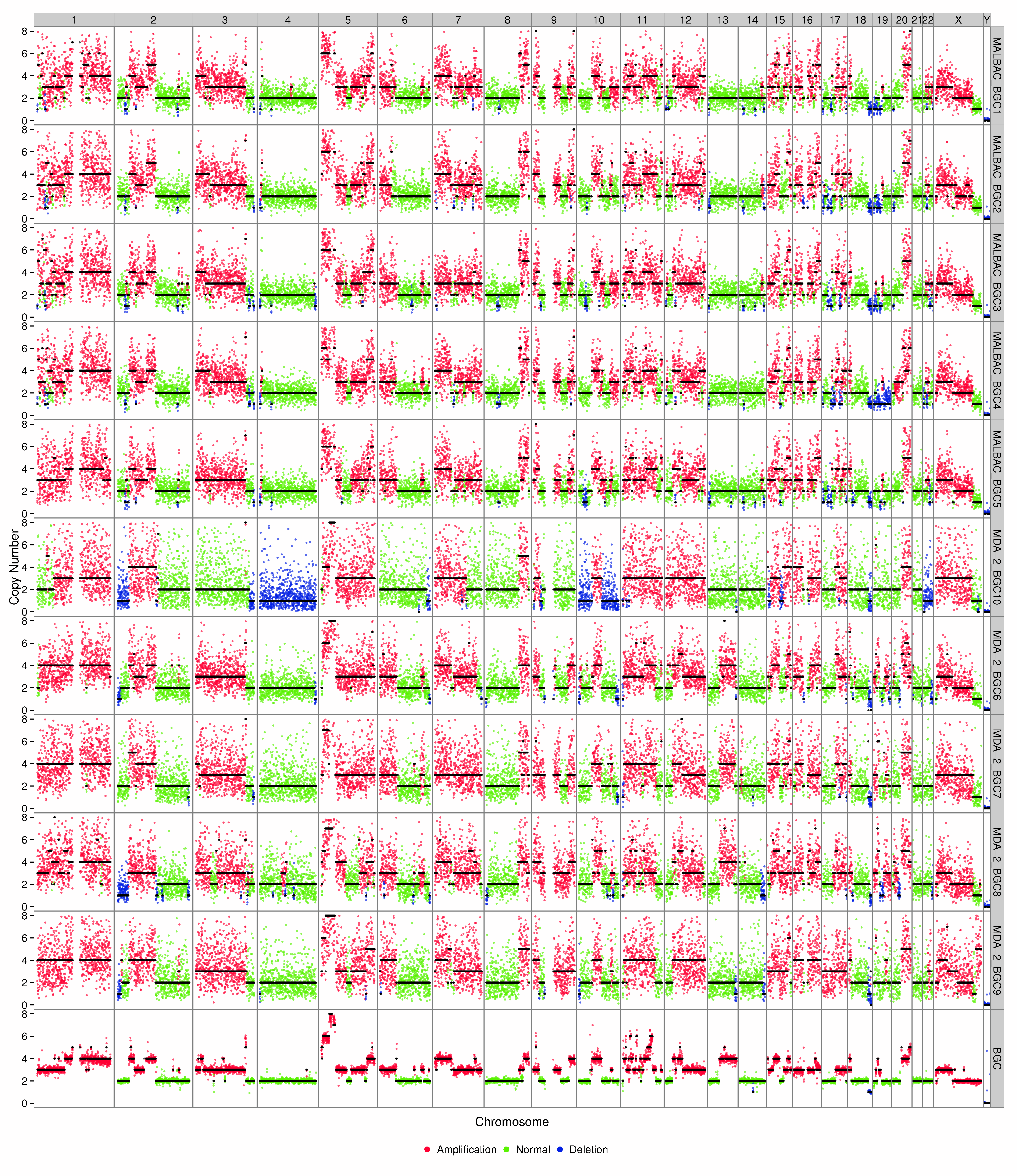

Supplement: Additional file 24: Figure S11b. — The landscape of CNVs of BGC823 single cells amplified by MALBAC or MDA-2. These BGC823 single cells were sequenced on the LifeTech Ion Proton sequencer, and BGC823 bulk sequencing data is used as the unamplified control. We extracted ~0.1X data from both the BGC823 single cells and the bulk to detect CNVs. [file 13742_2015_68_MOESM24_ESM.png]
