# Supplementary material for: Comparison of variations detection between whole-genome amplification methods used in single-cell resequencing
Source: Gigascience. 2015 Aug 6;4:37. doi: 10.1186/s13742-015-0068-3 (PMC4527218; doi:10.1186/s13742-015-0068-3)
Supplement: Additional file 12: — Supplementary Note. The performance comparison of mitochondrial genome assembly between MDA and MALBAC. [file 13742_2015_68_MOESM12_ESM.docx]

**Additional file 12: Supplementary Note**

In our study, we focused on reference-based resequencing and variations detection of single cells derived from human and cancer cell lines. However, we also tried to assess the influence of MDA and MALBAC on assembly (we excluded the deep sequencing data of the DOP-PCR method because of its limitation of the genome coverage). Nevertheless, the 30X whole genome sequencing dataset has insufficient depth to perform high-quality whole-genome assembly [[1](#_ENREF_1)]. Instead, we chose the mitochondrial genome to survey the performance of the assembly from single-cell data generated by MDA and MALBAC.

To compare the mitochondrial genome assembly performance between MDA and MALBAC methods, we first extracted the read-pairs that could be mapped to human mitochondrial genome for each sample. We then performed sequencing errors correction and *de novo* assembly using *SPAdes* [[2](#_ENREF_2)] (v3.5.0) with default parameters (MDA-amplified data with parameter ‘--sc’, which is required specifically for MDA data). Finally, we calculated the N50 and total length of assembled contig sequence for each sample.

We measured the total length and N50 of contigs to evaluate the assembly quality in the following **Table SN**. We observed that the total length of contigs for all mitochondrial genomes, and the N50 of contigs for most mitochondrial genomes, could achieve ~16 kb, approaching the total length of the whole mitochondrial genome [[3](#_ENREF_3)]. This indicated that the mitochondrial assembly ability and quality of MDA and MALBAC may be comparable, consistent with the previous report [[4](#_ENREF_4)]. However, we found two single cells amplified by MALBAC that showed lower N50 of contigs (3,194 and 9,163, respectively), revealing that MALBAC-generated data may have poor stability on assembly.

The N50 of contigs for most mitochondrial genomes amplified by MALBAC and MDA could reach the total length of the mitochondrial genome, demonstrating that the small size of the mitochondrial genome may inadequately reflect the variances of assembly contig length between two WGA methods. In addition, the high copy numbers [[5](#_ENREF_5)] and haplotype characteristics [[6](#_ENREF_6)] of mitochondrial genome may lead to the elimination of the random WGA impacts introduced by different WGA methods by the read error correction before the mitochondrial genome assembly. In other words, the small size and high copy numbers of the mitochondrial genomes may narrow the gap of assembly quality between MALBAC and MDA amplified data, and thus limit the extent to which comparison of mitochondrial assembly performance between them is possible. Despite these limitations, MALBAC may have comparable assembly quality as MDA but lower stability of the assembly than MDA by mitochondrial assembly.

| **Table SN. A Comparison of MtDNA Assembly Performance between MDA and MALBAC Amplified Data.** | | | | | |
| --- | --- | --- | --- | --- | --- |
| Sequence platform | WGA method | Read length | Sample index | Contig N50 | Contig total length |
| Illumina sequencer | MALBAC | 55 | SW480-1 | 16,565 | 16,565 |
|  | MALBAC | 55 | SW480-2 | 16,604 | 16,604 |
|  | MALBAC | 65 | SW480-3 | 3,194 | 16,170 |
|  | MALBAC | 65 | SW480-4 | 9,163 | 15,258 |
|  | MALBAC | 65 | SW480-5 | 16,309 | 16,309 |
|  | - | 90 | SW480-HEC | 16,626 | 16,626 |
|  | - | 100 | SW480-SCD | 16,627 | 16,627 |
|  | MDA-3 | 100 | MDA-3_45 | 16,592 | 16,592 |
|  | MDA-2 | 100 | MDA-2_46 | 16,630 | 16,630 |
|  | MDA-2 | 100 | MDA-2_47 | 16,585 | 16,585 |
|  | MDA-2 | 100 | MDA-2_66 | 16,573 | 16,573 |
|  | MDA-2 | 100 | MDA-2_M16 | 16,601 | 16,601 |
|  | MDA-2 | 100 | MDA-2_M6 | 16,592 | 16,592 |
|  | - | 100 | YH-mix | 16,630 | 16,630 |

**References**

1. Li R, Zhu H, Ruan J, Qian W, Fang X, Shi Z, *et al*. De novo assembly of human genomes with massively parallel short read sequencing. Genome Res. 2010;20:265-72. doi:10.1101/gr.097261.109.

2. Bankevich A, Nurk S, Antipov D, Gurevich AA, Dvorkin M, Kulikov AS, *et al*. SPAdes: a new genome assembly algorithm and its applications to single-cell sequencing. J Comput Biol. 2012;19:455-77. doi:10.1089/cmb.2012.0021.

3. Anderson S, Bankier AT, Barrell BG, de Bruijn MH, Coulson AR, Drouin J, et al. Sequence and organization of the human mitochondrial genome. Nature. 1981;290:457-65.

4. de Bourcy CF, De Vlaminck I, Kanbar JN, Wang J, Gawad C, Quake SR. A quantitative comparison of single-cell whole genome amplification methods. PloS One. 2014;9:e105585. doi:10.1371/journal.pone.0105585.

5. Clay Montier LL, Deng JJ, Bai Y. Number matters: control of mammalian mitochondrial DNA copy number. J Genet Genom = Yi chuan xue bao. 2009;36:125-31. doi:10.1016/S1673-8527(08)60099-5.

6. Castro JA, Picornell A, Ramon M. Mitochondrial DNA: a tool for populational genetics studies. Int Microbiol. 1998;1:327-32.
