# Supplementary material for: Comparison of variations detection between whole-genome amplification methods used in single-cell resequencing
Source: Gigascience. 2015 Aug 6;4:37. doi: 10.1186/s13742-015-0068-3 (PMC4527218; doi:10.1186/s13742-015-0068-3)
Supplement: Additional file 16: Figure S7. — A schematic of one chimeric ITX calling within WDHD1 gene in MDA-2_M6. The breakpoints and supporting reads of the chimeric ITX are shown. [file 13742_2015_68_MOESM16_ESM.pdf]

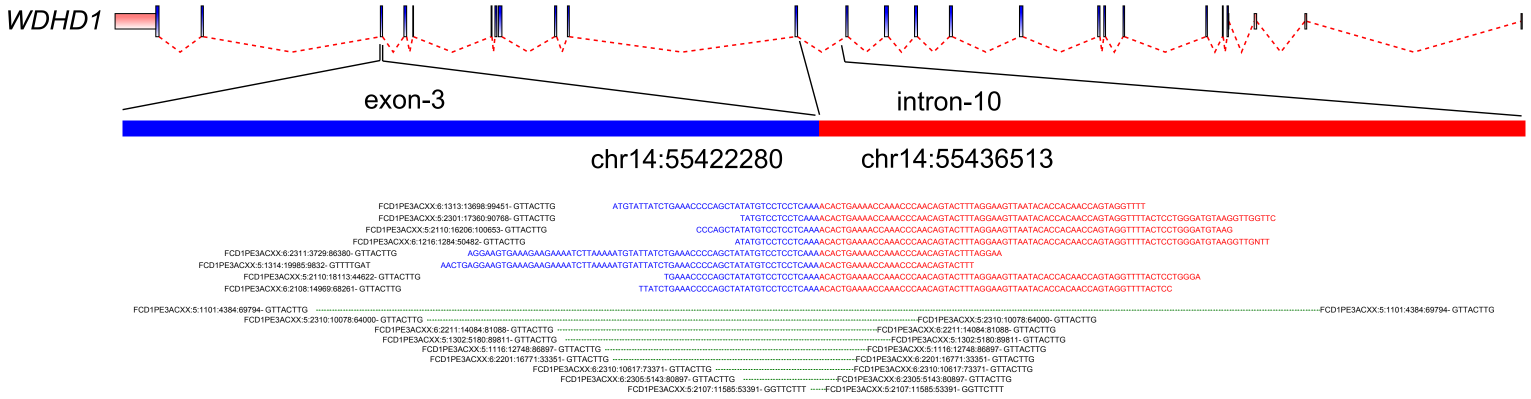
WDHD1
exon-3
intron-10
chr14:55422280
chr14:55436513
