# Supplementary material for: Comparison of variations detection between whole-genome amplification methods used in single-cell resequencing
Source: Gigascience. 2015 Aug 6;4:37. doi: 10.1186/s13742-015-0068-3 (PMC4527218; doi:10.1186/s13742-015-0068-3)
Supplement: Additional file 17: Figure S8. — A schematic of one chimeric CTX calling between chromosome 1 and 14 in MDA-2_47. The breakpoints and supporting reads of the chimeric CTX are shown. [file 13742_2015_68_MOESM17_ESM.pdf]

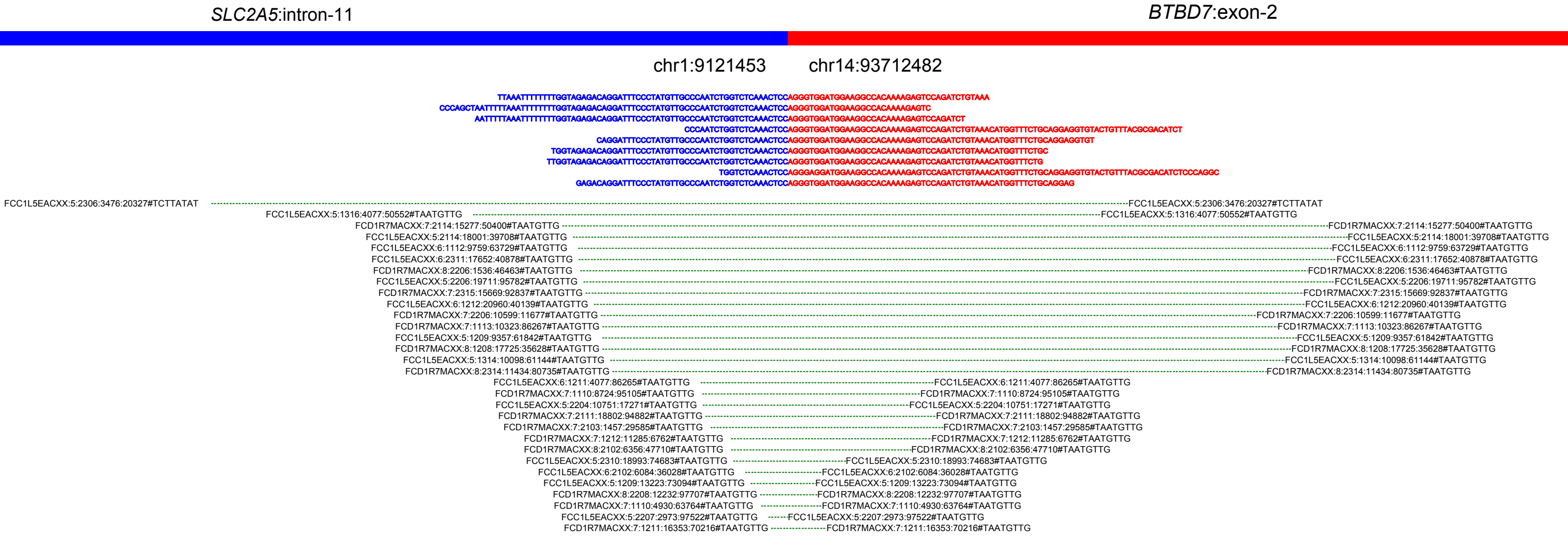

SLC2A5:intron-11
BTBD7:exon-2
chr1:9121453
chr14:93712482
TTAAATTTTTTTTGGTAGAGACAGGATTTCCCTATGTTGCCCAATCTGGTCTCAAACTCCAGGGTGGATGGAAGGCCACAAAAGAGTCCAGATCTGTAAA
CCCAGCTAATTTTTAAATTTTTTTTGGTAGAGACAGGATTTCCCTATGTTGCCCAATCTGGTCTCAAACTCCAGGGTGGATGGAAGGCCACAAAAGAGTC
AATTTTTAAATTTTTTTTGGTAGAGACAGGATTTCCCTATGTTGCCCAATCTGGTCTCAAACTCCAGGGTGGATGGAAGGCCACAAAAGAGTCCAGATCT
CCCAATCTGGTCTCAAACTCCAGGGTGGATGGAAGGCCACAAAAGAGTCCAGATCTGTAAACATGGTTTCTGCAGGAGGTGTACTGTTTACGCGACATCT
CAGGATTTCCCTATGTTGCCCAATCTGGTCTCAAACTCCAGGGTGGATGGAAGGCCACAAAAGAGTCCAGATCTGTAAACATGGTTTCTGCAGGAGGTGT
TGGTAGAGACAGGATTTCCCTATGTTGCCCAATCTGGTCTCAAACTCCAGGGTGGATGGAAGGCCACAAAAGAGTCCAGATCTGTAAACATGGTTTCTGC
TTGGTAGAGACAGGATTTCCCTATGTTGCCCAATCTGGTCTCAAACTCCAGGGTGGATGGAAGGCCACAAAAGAGTCCAGATCTGTAAACATGGTTTCTG
TGGTCTCAAACTCCAGGGAGGATGGAAGGCCACAAAAGAGTCCAGATCTGTAAACATGGTTTCTGCAGGAGGTGTACTGTTTACGCGACATCTCCCAGGC
GAGACAGGATTTCCCTATGTTGCCCAATCTGGTCTCAAACTCCAGGGTGGATGGAAGGCCACAAAAGAGTCCAGATCTGTAAACATGGTTTCTGCAGGAG
FCC1L5EACXX:5:2306:3476:20327#TCTTATAT FCC1L5EACXX:5:2306:3476:20327#TCTTATAT
FCC1L5EACXX:5:1316:4077:50552#TAATGTTG FCC1L5EACXX:5:1316:4077:50552#TAATGTTG
FCD1R7MACXX:7:2114:15277:50400#TAATGTTG FCD1R7MACXX:7:2114:15277:50400#TAATGTTG
FCC1L5EACXX:5:2114:18001:39708#TAATGTTG FCC1L5EACXX:5:2114:18001:39708#TAATGTTG
FCC1L5EACXX:6:1112:9759:63729#TAATGTTG FCC1L5EACXX:6:1112:9759:63729#TAATGTTG
FCC1L5EACXX:6:2311:17652:40878#TAATGTTG FCC1L5EACXX:6:2311:17652:40878#TAATGTTG
FCD1R7MACXX:8:2206:1536:46463#TAATGTTG FCD1R7MACXX:8:2206:1536:46463#TAATGTTG
FCC1L5EACXX:5:2206:19711:95782#TAATGTTG FCC1L5EACXX:5:2206:19711:95782#TAATGTTG
FCD1R7MACXX:7:2315:15669:92837#TAATGTTG FCD1R7MACXX:7:2315:15669:92837#TAATGTTG
FCC1L5EACXX:6:1212:20960:40139#TAATGTTG FCC1L5EACXX:6:1212:20960:40139#TAATGTTG
FCD1R7MACXX:7:2206:10599:11677#TAATGTTG FCD1R7MACXX:7:2206:10599:11677#TAATGTTG
FCD1R7MACXX:7:1113:10323:86267#TAATGTTG FCD1R7MACXX:7:1113:10323:86267#TAATGTTG
FCC1L5EACXX:5:1209:9357:61842#TAATGTTG FCC1L5EACXX:5:1209:9357:61842#TAATGTTG
FCD1R7MACXX:8:1208:17725:35628#TAATGTTG FCD1R7MACXX:8:1208:17725:35628#TAATGTTG
FCC1L5EACXX:5:1314:10098:61144#TAATGTTG FCC1L5EACXX:5:1314:10098:61144#TAATGTTG
FCD1R7MACXX:8:2314:11434:80735#TAATGTTG FCD1R7MACXX:8:2314:11434:80735#TAATGTTG
FCC1L5EACXX:6:1211:4077:86265#TAATGTTG FCC1L5EACXX:6:1211:4077:86265#TAATGTTG
FCD1R7MACXX:7:1110:8724:95105#TAATGTTG FCD1R7MACXX:7:1110:8724:95105#TAATGTTG
FCC1L5EACXX:5:2204:10751:17271#TAATGTTG FCC1L5EACXX:5:2204:10751:17271#TAATGTTG
FCD1R7MACXX:7:2111:18802:94882#TAATGTTG FCD1R7MACXX:7:2111:18802:94882#TAATGTTG
FCD1R7MACXX:7:2103:1457:29585#TAATGTTG FCD1R7MACXX:7:2103:1457:29585#TAATGTTG
FCD1R7MACXX:7:1212:11285:6762#TAATGTTG FCD1R7MACXX:7:1212:11285:6762#TAATGTTG
FCD1R7MACXX:8:2102:6356:47710#TAATGTTG FCD1R7MACXX:8:2102:6356:47710#TAATGTTG
FCC1L5EACXX:5:2310:18993:74683#TAATGTTG FCC1L5EACXX:5:2310:18993:74683#TAATGTTG
FCC1L5EACXX:6:2102:6084:36028#TAATGTTG FCC1L5EACXX:6:2102:6084:36028#TAATGTTG
FCC1L5EACXX:5:1209:13223:73094#TAATGTTG FCC1L5EACXX:5:1209:13223:73094#TAATGTTG
FCD1R7MACXX:8:2208:12232:97707#TAATGTTG FCD1R7MACXX:8:2208:12232:97707#TAATGTTG
FCD1R7MACXX:7:1110:4930:63764#TAATGTTG FCD1R7MACXX:7:1110:4930:63764#TAATGTTG
FCC1L5EACXX:5:2207:2973:97522#TAATGTTG FCC1L5EACXX:5:2207:2973:97522#TAATGTTG
FCD1R7MACXX:7:1211:16353:70216#TAATGTTG FCD1R7MACXX:7:1211:16353:70216#TAATGTTG
